# Science at the Secretariat of the International Seabed Authority: post-normal science for recalibration of policy instruments.

**DOI:** 10.12688/openreseurope.18879.3

**Published:** 2025-06-30

**Authors:** Aistė Klimašauskaitė, Paul Dees, Silvio Oscar Funtowicz, Laura Elisabet Drivdal, Ola G. Berta

**Affiliations:** 1 Centre for the Study of the Sciences and the Humanities, University of Bergen, Bergen, Hordaland, Norway; 2 Bjerknes Centre for Climate Research, University of Bergen, Bergen, 5020, Norway; 3 Geophysical Institute, University of Bergen, Bergen, 5007, Norway; 4 University of Bergen Library, University of Bergen, Bergen, 5007, Norway; 5 Department of Social Anthropology, University of Bergen, Bergen, 5007, Norway

**Keywords:** International Seabed Authority, post-normal science, marine science, deep-seabed mining, deep-sea mining, epistemic severing, epistemic repair.

## Abstract

Post-normal conditions require post-normal resolutions. When stakes are high and values are disputed, policy discussions and decisions cannot rely on science alone. Such discussions require *post-normal science*, which centralizes the qualitative value of diverse knowledge groups. In this essay we discuss the usefulness of post-normal science in the context of the Secretariat’s work at the International Seabed Authority. In particular, we explore the need to expand the understanding of science and knowledge – key components informing deep-seabed policies. Marine science encompasses a broad range of research topics and interests – from blue humanities with analyses of human-Ocean relations, to physics of particulate matter, to marine policy research. The growing number of disciplines applied to marine science demonstrates our increasing collective knowledge of the Ocean and, in turn, our appreciation of knowledge limitations. Such knowledge challenges are discussed among natural and social scientists. The Secretariat depends on broad expertise, from natural sciences, social sciences, civil society and beyond. In sum, our thesis is the following: we argue that *post-normal science* can help metaphorically recalibrate policy-shaping instruments (the practice) so that decision-making processes and policy agendas are implemented for transformative actions.

## Plain language summary

Decisions regarding current, pressing environmental challenges require collaboration among local communities, governments, and scientists. However, such collaborations explicitly or implicitly prioritize the knowledge of scientists rather than locals. We suggest that such prioritization is problematic, and we here present the post-normal science approach as a possible remediation from the philosophy of science. We focus on the Secretariat of the International Seabed Authority as one important place where intergovernmental decisions are made regarding deep-seabed mining in the Pacific Ocean. We argue that the Secretariat has the power to nurture and facilitate broad collaborations, but often falls short. This is therefore an opportunity to learn from the post-normal science approach, and to improve work done at the Secretariat. We start the essay with an introduction to current developments at the International Seabed Authority. We then present the role of the Secretariat and how it impacts decisions made at the International Seabed Authority. We describe post-normal science and its relevance and uses in the policy context (where decisions are made). Finally, we show one example of how post-normal science could be used in the work of the Secretariat.

## Abstract (it)

Le condizioni post-normali richiedono risoluzioni post-normali. Quando le poste in gioco sono alte e i valori sono in discussione, i dibattiti e le decisioni politiche non possono basarsi solo sulla scienza. In tali casi, discussioni e scelte devono fare riferimento alla scienza post-normale e riconoscere che diversi individui e molteplici gruppi possono apportare un utile contributo di conoscenze. Discutiamo dell'utilità della scienza post-normale nel contesto del lavoro del Segretariato dell'Autorità Internazionale per i Fondali Marini. In particolare, esploriamo la necessità di per scienza e conoscenza, i componenti chiave che informano le politiche relative ai fondali marini profondi. La scienza marina comprende una vasta gamma di argomenti e interessi di ricerca: dalle scienze umane blu, con l’analisi delle relazioni umanità-oceano, alla fisica delle particelle fino alla ricerca sulle politiche marine. Il crescente numero di discipline applicate alla scienza marina dimostra un aumento della nostra conoscenza collettiva sull’oceano e, di conseguenza, una maggiore consapevolezza e ammissione dei limiti di ciò che sappiamo. Queste sfide conoscitive vengono discusse tra scienziati naturali e sociali. Il Segretariato si avvale di un ampio spettro di competenze provenienti dalle scienze naturali, le scienze sociali, la società civile e oltre. In sintesi, sosteniamo che la può contribuire a ricalibrare metaforicamente gli strumenti che modellano le politiche (la pratica), affinché i processi decisionali e le agende politiche siano implementate per azioni trasformative.

## Abstract (es)

Condiciones posnormales requieren resoluciones posnormales. Cuando los riesgos son elevados y los valores están en disputa, las discusiones y decisiones políticas no pueden depender únicamente de la ciencia. Este tipo de debates requiere una ciencia posnormal, que pone énfasis en el valor cualitativo de diversos tipos de conocimiento. En este artículo, discutimos la utilidad de la ciencia posnormal en el contexto de las actividades de la Secretaría de la Autoridad Internacional de los Fondos Marinos. En particular, exploramos la necesidad de ampliar el concepto de ciencia y conocimiento, componentes clave que sustentan las políticas relacionadas con los fondos marinos profundos. La ciencia marina abarca una amplia gama de temas de investigación e intereses: desde las humanidades azules, que analizan las relaciones entre los seres humanos y el océano, hasta la física de partículas y los estudios sobre políticas marinas. El creciente número de disciplinas aplicadas a la ciencia marina refleja un aumento en nuestro conocimiento colectivo del océano y, a su vez, un reconocimiento y una apreciación cada vez mayores de las limitaciones de lo que sabemos. Estos desafíos epistemológicos se abordan tanto en las ciencias naturales como en las sociales. La Secretaría depende de una amplia variedad de conocimientos que provienen de las ciencias naturales, las ciencias sociales, la sociedad civil y otros sectores. En resumen, sostenemos que la ciencia posnormal puede contribuir a recalibrar metafóricamente los instrumentos que moldean las políticas (la práctica), permitiendo que los procesos de toma de decisiones y las agendas políticas se transformen en acciones verdaderamente transformadoras.

## Introduction

Since its establishment in 1994, multiple introductions and texts have been written to analyze the International Seabed Authority (ISA) and its work, negotiations, and mandate (for recent examples, see Blanchard *et al.*, 2023; Bosco *et al.*, 2023; Pickens *et al.*, 2024; Singh, 2022). ISA was born out of the United Nations Convention on the Law of the Sea (UNCLOS). It is the authority that regulates activities in the international seabed. The key legal principle in the UNCLOS Article 136 regarding the international seabed – *the Area* (which includes the seabed, Ocean floor and subsoil thereof) – is that it is the *common heritage of [hu]mankind.* Initially, this legal principle was meant to steer any future seabed exploitation for the benefit of all humanity ( Massimi, 2024; Meyer, 2022; Ranganathan, 2019). Today, however, multiple international Ocean negotiations demonstrate how contested the interpretation of this key principle is. Massimi (2024) argues that one of such interpretations is rooted in epistemic injustice. In part, this interpretation flows from the main activity in the international seabed – industrial deep-sea mining (DSM) exploration. In this essay, we wish to introduce the debate around and within the ISA to a diverse audience. We therefore clarify that the phrases "deep-sea mining" and "deep-seabed mining" are used interchangeably in academic and policy texts. We will use the abbreviated DSM throughout the remainder of the essay.

Out of a multitude of activities possible on the international seabed, it is not accidental that exploration for mining is concentrated most on today. Ranganathan (2019, pp. 576) tells how the history of UNCLOS paved the way for today’s industrial interests – “the law’s constitutive effects” in normalizing “an extractive imaginary of the ocean floor.” In sum, ISA embodies a clash of private interests with public interests and a contradictory mandate to protect and exploit ( Holst, 2023). We therefore propose a policy-relevant framework to be used when stakes are high, and values are contested.

Today, under ISA’s authority, the majority of deep-sea mining exploration activities are concentrated in the Clarion Clipperton Zone (CCZ). Ecosystem-based management is an integral component of the Environmental Management Plan in the CCZ ( Guilhon *et al.*, 2021; ISA, 2011). Yet, no similar approach or framework exists around policy discussions at the ISA. Current rhetoric from the Secretariat – ISA’s administrative body – indicates a science-based approach (e.g., ISA, 2024). In turn, we describe and discuss post-normal science (PNS), which, we argue, expands the science-based approach and gives a deeper meaning to the key principle of the *common heritage of [hu]mankind.* Our focus is on the Secretariat, which has a key role in policy shaping at the ISA (described in greater detail in the following section).

One aspect that we highlight as crucial for an open debate is the responsibility of informing decisions about *the Area –* the policy-shaping space. In a recent World Oceans Day 2023 statement titled “Celebrating women scientists advancing deep-sea research,” Mr. Michael W. Lodge (2023), the soon-former Secretary-General ^1^ at ISA, wrote:

*…ISA has the exclusive responsibility to promote and encourage marine scientific research in the Area and to coordinate the dissemination of the results. (…)*
*The science undertaken and the data and information collected directly inform global decision-making processes and policy agendas for transformative actions.*

The quote highlights the key role of marine natural and life sciences. Yet, marine science encompasses a broad range of research topics and interests – from blue humanities with analyses of human-Ocean interactions, to Ocean optics and marine policy research. The concerted combination of all this knowledge shows how far we have come to know the Ocean. In turn, we have realized growing limitations to this knowledge – a broader reflection which must be grappled with in the context of the industrial expansion in the Ocean (sometimes called the blue growth or the *blue acceleration* ( Jouffray *et al.*, 2021)). The knowledge challenges discussed by natural and social scientists include physical and philosophical aspects of human expansion into the deep sea ( Amon *et al.*, 2022; Germond-Duret, 2022; Smith *et al.*, 2020). Acknowledging the limits of human understanding and appreciation of the Ocean calls for broad expertise and participation from natural sciences, social sciences, civil society and beyond ( Escobar *et al.*, 2021; Tilot *et al.*, 2021b). There is a broad agreement that science alone is not enough – a vibrant academic discussion since the 90s (which we refer to and acknowledge later in this introduction, and the chapter titled “The post-normal science lens”).

The above quote from Mr. Lodge is also an example of ISA’s mandate in action, where the UNCLOS text needs to be shaped into a workable deep-seabed governance framework. The specific word “science”, and its associations and meanings as they appear in the UNCLOS text, are important. For example, “research” appears 162 times, “marine scientific research” appears 101 times and “science” is mentioned a total of 15 times, including 14 mentions of “marine science.” Yet, UNCLOS does not define what it means by research, science or marine science, nor does it outline the quality features of these terms. The only allusions to quality are the mentions of “appropriate scientific criteria” (Article 201), “appropriate scientific methods” (Article 240) or “recognized scientific methods” (Article 165 and Article 204), and “best available scientific evidence” (Article 234). This inadvertently benefits those who represent the Western notion of science and scientific methods, which often excludes other types of knowledge, as Massimi (2024) describes:

*As a result, marine scientific research understood as per UNCLOS Art 242–244 and again under the BBNJ Treaty Art. 40–42 on capacity-building and transfer of marine technology risks becoming an ‘epistemic trademark’ to designate de facto varieties of non-oral and nonlocal knowledge that are typically the repository of Global North lab facilities and databanks—i.e. the kind of knowledge that requires technological infrastructures, human resources and institutional facilities to be carried out on a large commercial scale, and which typically tends to be seen as being ’disseminated’ to other ’developing countries’ under the deficit model.*

Broad knowledge is a key to quality decisions. Science alone is not enough when informing policies where stakes are high and values are in dispute ( Funtowicz & Ravetz, 2020). In the same way that ecosystem-based management is used as a framework to provide structure to deep-seabed governance and management, we suggest *post-normal science* as a framework for informing policy-shaping with a focus on *knowledge ecosystems*. Post-normal science centralizes transdisciplinarity through an extended peer community of diverse knowledge holders – a knowledge ecosystem.

A knowledge ecosystem combines different *knowledges* or different ways of knowing ( Haraway, 2013). Since the Enlightenment, the widely accepted way of knowing something in Western academic circles has been to employ the scientific method. To be ready to accept *knowledges* is to be cognizant of knowledge communities for which the scientific method is not the only method of discovery. PNS should therefore not be used as a replacement for the scientific method, but rather as a method of informing policy discussions with a range of *knowledges*, including traditional local knowledge. In this commentary, we focus on the Secretariat as one of the main bodies steering the policy discussions at ISA. We stress that PNS is relevant to other governmental and intergovernmental bodies as well. The question of knowledge has been explored in the context of the Intergovernmental Panel on Climate Change (IPCC) and the Intergovernmental Science-Policy Platform on Biodiversity and Ecosystem Services (IPBES) ( Borie *et al.*, 2021), as well as the International Council for the Exploration of the Sea (ICES) ( Kim & Dankel, 2025). Yet, knowledge use at ISA has historically attracted less attention and will be the focus of our commentary. We suggest that PNS can help metaphorically recalibrate the policy practice to implement decision-making processes and policy agendas for transformative – *just* – actions. However, this paper is not an empirical study and the reader should be advised that observations of the ISA Secretariat’s work are purposive and not systematic. We show that for the main argument, these observations are informative and sufficient.

We start the essay with an overview of the role of ISA’s Secretariat in policy-shaping through various activities such as commissioning of studies. We then shortly present PNS. Finally, we choose one example of how PNS could be used at the Secretariat. The example is focused on one ecosystem valuation report that was commissioned by ISA’s Secretariat. We emphasize that this is a context specific example and suggests only one way of approaching the question of knowledge in policy space through the PNS lens.

## The Secretariat as a key body in policy-shaping at ISA

The Secretariat is a complex and central administrative body which shapes policy discussions at the ISA through various responsibilities and activities. In a direct policy-shaping context, it oversees production of various reports informing the work of ISA bodies with policy decision-making power ( Bosco *et al.*, 2023). For example, the Secretariat informs key policy discussions such as *benefit sharing* ( Wilde *et al.*, 2023). (Note that, current discussions regarding benefit sharing include criticism about the potential “insignificant” monetary benefits and alternative solutions that would lead to indirectly paying for mining impacts, rather than any meaningful benefits to humankind ( Wilde *et al.*, 2023).) These responsibilities of the Secretariat are also presented on ISA’s web page ( ISA, 2023c):

*Producing reports and other documents containing information, analyses, historical background, research findings, policy suggestions, etc. that facilitate the deliberations and decision-making by the other principal organs and their subsidiary bodies.*

We note that Bosco and colleagues (2023) highlight the conflictual meaning of facilitation:

*While in any one case the line between facilitating another organ’s work and taking on its responsibilities is hard to pinpoint, the Secretariat’s disproportionate output is striking: on average, from the first to 26th sessions the Secretariat authored about 56% of the ISA’s reports and about half of its official documents overall.*

Independent reports regarding the Secretariat’s work indicate the wide technical and non-technical impact it has on the ISA’s structure, including the Legal and Technical Commission (LTC) and the Finance Committee (FC) ( Billett *et al.*, 2023; Bosco *et al.*, 2023). For example, Bosco and colleagues (2023) summarize: ‘One notable trend in the ISA’s evolution is the Secretariat’s assumption of work seemingly designated to the deliberative bodies (the Assembly, Council, LTC, and FC).’ More importantly, they warn that ‘…overreliance on the Secretariat detracts from member states’ ability to drive policy’ ( Bosco *et al.*, 2023). Among such activities, the Secretariat seems to control information flows within ISA ( Bosco *et al.*, 2023):

*It is largely left to the Secretariat to determine what information is confidential and what will be shared with the other organs. While the LTC has more access to confidential information than the other organs, it is not allowed to distribute information without the Secretariat’s consent.*

In an indirect policy-shaping context, the Secretariat promotes some policy-relevant questions. For example, Mr. Lodge (2020) presented mining the Pacific seabed as a necessity for the energy transition. Yet, social science contributions to energy research do not seem to fall under the Secretariat’s definition of marine science. The Secretariat does not appear to commission any social science reports on the topic of energy transition, which results in studies by science and technology studies researchers, human geographers, sociologists, and others ( Korsnes *et al.*, 2023; Riofrancos *et al.*, 2023) being regarded as an irrelevant or unneeded group of expertise. The Secretary-General still connected ISA’s work to the broader international political and research context such as energy research while omitting works from a wide range of expertise and knowledge. The Secretariat has thus inadvertently isolated its work from a valuable section of academia. We reiterate that it is the Secretariat that often decides what the relevant expertise for ISA is ( Bosco *et al.*, 2023):

*Conflicts of interest also complicate the ISA’s use of consultants to create reports and advise on policy. The Secretariat employs consultants on a contractual basis for many tasks to cope with its high workload and relatively thin staffing. However, consultants are selected and contracts signed without formal input from any of the other organs, leaving members and the public unable to assess the impartiality and credentials of consultants.*

In a different layer of policy shaping, the Secretariat controls the physical participation of observers at ISA’s meetings ( ISA, 2023b) and oversees engagement with the public and the science community ( Ardron *et al.*, 2023; ISA, 2018; ISA, 2023a). The Secretariat defines the rules of participation that directly affect the observers ( ISA, 2023b) and, in part, shapes who is represented and who has a voice. Indigenous peoples are still underrepresented at the table ( Earth Negotiations Bulletin, 2023; Morgera & Lily, 2022). When they are represented, there is a set of obstacles to acknowledge. For example, writing about Solomon Pili Kaho‘ohalahala’s speech at the ISA’s meeting in 2023, Ranganathan (2024) highlights the fact that many inhabitants of Oceania enter ISA’s meetings under the assumed limits and logic of the Ocean as an industrial site, as opposed to being a *home* for the diverse local communities. That is, the Pacific Islanders are required to explain the obvious meaning of *their home* to those who see and perceive the Pacific Ocean as a potential mining site ( Ranganathan, 2024). Being *home*, however, does not seem to match the value of being a potential mining site as seen by some of the policymakers and experts associated with the ISA. *Home* does not seem to convey expertise and power – not the technological expertise and financial capabilities that are held by those engaged in exploration in the CCZ.

Unsurprisingly, this echoes the colonial logic of viewing the Global South ^2^ as a space for extraction. This misalignment goes as high as the discussions on benefit sharing in the Secretariat. For example, Mr. Lodge and colleagues (2017) discuss the lack of clarity on *if* and *how* ISA could implement benefit sharing with Indigenous peoples, without acknowledging the larger context of existing benefits – the Pacific Ocean is a home for its inhabitants before it is a mining site for the selected few. Further, considering local contributions and participation under deeply rooted constraints, Ranganathan (2024, pp. 91) points out:

*This does not mean that there is nothing for them to articulate, but it places them under a dual burden: first, of making their histories and epistemologies comprehensible, thus a burden of translation; second, of formulating demands that can be comprehended within pre-defined parameters, thus a burden of accommodation. In addition, of course, is the burden of finding paths to present their demands to the ISA, absent formal participatory mechanisms. What then happens, when they seek to exceed these terms, such as in calling for a ban on seabed mining?*

In social and natural science scholarly work, traditional knowledge in Oceania is recognized on its own merits and not as something with lesser value than scientific knowledge (e.g., Bode & Yarina, 2020; Escobar *et al.*, 2021; Mulalap *et al.*, 2020; Tilot *et al.*, 2021a). Yet, the international seabed policy context seems to be still dominated by a narrow understanding of what “relevant” knowledge and, in turn, science is ( Massimi, 2024). So, even if diverse participation at ISA is increasing, the current methods of managing the complex discussions regarding the international seabed policies seemingly fall short of implementing the ideals of UNCLOS’ key principle of *the common heritage of [hu]mankind*. Therefore, in the next sections, we will describe PNS as a policy practice and mindset. We suggest that PNS approaches mirror the stated aspirations of UNCLOS: seeking to project and bolster the *common heritage of [hu]mankind* as a core idea for a better future.

## The post-normal science lens

Since the 1990s, PNS scholars have called for greater transdisciplinarity and *extended peer communities* of scientists, policymakers, and society to work together ( Funtowicz & Ravetz, 1993; Funtowicz & Ravetz, 2020; Kovacic *et al.*, 2019). When translating scientific findings to policy, the complexity, uncertainty, ambiguity, and values implicit in the original research can be overlooked (e.g., Saltelli *et al.*, 2020). The quality of policy discussions and decisions could be improved through the acknowledgement of potential biases and the exploration of alternative choices all with the help of diverse knowledge holders and sources. It is important to note that PNS is not an alternative to science or a different kind of science, but rather different conditions of the science for policy context ( Saltelli *et al.*, 2020). It is a framework by which science informs policy under complex conditions. Reaching quality outcomes in such situations requires rich knowledge from diverse sources. In sum, PNS could be seen as a *knowledge ecosystem-based management*.

PNS is applicable in situations ‘where facts are uncertain, values in dispute, stakes high and decisions urgent’ ( Funtowicz & Ravetz, 2020). Current debates about the international seabed at ISA exemplify a situation where scientific uncertainty and stakes are high ( Amon *et al.*, 2022), with a growing body of valuable works discussing environmental and regulatory uncertainties ( Ginzky *et al.*, 2020; Haeckel *et al.*, 2020; Pickens *et al.*, 2024; Roest *et al.*, 2023; Wilde *et al.*,2023). If deep-sea mining in the Area is permitted, even at a limited scale to explore the viability of the industry, the historical DISCOL project and a multitude of other studies that followed have revealed there is a risk of irrevocable damage to complex systems (e.g., Jones *et al.*, 2017; Simon-Lledó *et al.*, 2019; Vonnahme *et al.*, 2020). Conflicting values are debated by various civil society groups, politicians, and scientific community groups ( Kim, 2017). Finally, a sense of urgency concerning these decisions is apparent, as the Republic of Nauru (the sponsoring state of the Metals Company) has ‘opted to invoke section 1(15) and requested that the [ISA] Council complete the elaboration and adoption of the [deep-sea mining] exploitation regulations by 9 July 2023’ ( Singh, 2022). While it did not lead to ISA issuing mining licenses without finalizing the regulations, this shows industry-constructed urgency. All three of the conditions detailed by Funtowicz and Ravetz (2020) are therefore present in the policy context at ISA. This indicates natural and social sciences alone are not enough for policymakers to make robust, quality decisions and that politicization of science (as shown by recent international Ocean governance debates ( De Santo *et al.*, 2019)) is unavoidable.

PNS invites policymakers to work within complexity, uncertainty, and a multiplicity of values through the extended peer community (see practice oriented special issue by Dankel *et al.*, (2017), including the contribution by Pereira and Saltelli (2017)). In PNS, the extended peer community consists ‘not merely of persons with some form or other of institutional accreditation (‘stakeholders’), but rather of all those with a desire to participate in the resolution of the issue’ ( Ravetz, 1999). PNS is not merely a different name for stakeholder consultation – it is a policy mindset and practice ( Funtowicz & Ravetz, 2020) where policy discussion input from parties like people from Oceania is as valuable as the input of marine engineers. In deep-sea mining, bias is often displayed in the choice of which experts are considered relevant or authoritative, and subsequently listened to – engineers and marine natural scientists are selected to be the voices of the deep sea more frequently than any other marine science experts ( Childs, 2022). Quality natural science studies are needed to guide policy in the deep seabed. Yet, natural science cannot guide some of the regulatory topics that are negotiated at ISA. For example, discussing options for *benefit sharing* ( Ranganathan, 2019) or the meanings of the *common heritage of [hu]mankind* ( Hunter *et al.*, 2018; Massimi, 2024) require acknowledging complex realities where even relying solely on social sciences is insufficient.

In part, various forms of public participation could exemplify one of the ways of extending the peer community (with their own benefits and pitfalls (e.g., Ballesteros & Dickey-Collas, 2023)). The UNCLOS PART XI. SECTION 2., and the agreement to consider the Area as the *common heritage of [hu]mankind*, define the public is a core beneficiary. ISA’s Secretariat has tried to involve this beneficiary through various initiatives, including the initiation of a deep-sea literacy action plan ( ISA, 2020). Yet, research shows that greater, more open communication with the public ( Morgera & Lily, 2022) and improved access to information ( Blanchard *et al.*, 2023) are still needed. In the context of UNCLOS, these should not be seen as marginal issues and challenges. Until now, we discussed examples within a limited scope and used PNS to broaden the limits of the discussions about the deep seabed. We think, however, it is helpful to take one example and explore it deeper. We do so in the following section.

## How PNS could be used at the Secretariat

In this section, we will explore how using the peer community – the diverse knowledge inside and outside of academia – can potentially help “recalibrate” policy-informing instruments at ISA’s Secretariat. We use the Secretariat’s commissioned “Report on the value of ecosystem services and natural capital of the Area” ( Brander & Guisado Goñi, 2023) as an example. We begin by acknowledging the guidance document associated with the report and the fact that it outlines various limitations of the report and its approaches, including the limitations of economic valuation studies (e.g., the controversy of commodification of nature) and the limitations of participatory scope that can be facilitated in any one study ( Brander, 2023). We also note that the report was reviewed by two people – one specializing in economics and one in extractive industries who, seemingly, were selected by the Secretariat ( Brander & Guisado Goñi, 2023, pp. 35). It is our opinion that the report and its review process are exemplary of how the Secretariat shapes content for policy inputs. As such, it is a useful example for applying the PNS lens to. Before we start, we anticipate one argument against routinely using the PNS lens when preparing reports is that doing so will require additional time and financing. Yet, we argue that as using PNS requires only engagement with the extended peer community and its knowledge, the likelihood of bias is reduced.

The following key findings in the report are our point of interest ( Brander & Guisado Goñi, 2023, pp. 32):

*Seabed habitats in the Area are recognised to provide a broad range of ecosystem services. Through consultations with 10 experts (8 organisations) conducted for this study, “key” ecosystem services that are of potentially high economic value were identified. These are genetic resources for use in medicines, biotechnology, and other industries; and the existence and bequest values that people hold for the preservation of unique biodiversity. Such results reflect the views of the limited number of experts consulted, and do not represent the full spectrum of potential views and perspectives from all ISA stakeholders.*
*The limited number of existing studies on the value of deep-sea ecosystem services are for study sites located in national EEZs and provide estimates for only four ecosystem services: food provisioning, genetic resources, carbon sequestration, and existence and bequest values. There are currently no studies that estimate values specifically for ecosystem services in the Area.*

We turn to the extended peer community and start with the most vivid issue that these findings exemplify: *epistemic severing* or the exclusion of knowledge. That is, the findings show that one specific knowledge community has “won” as their knowledge emerges as an indicator of ‘“key” ecosystem services that are of potentially “high economic value.” What does it mean to find ecosystem services that are “key” and of “high economic value”? We focus on the process of *finding*. The authors of the report, cited above, highlight the diversity of experts, their organizations and the literature on the topic of ecosystem valuation. However, we argue that what emerges is more hegemonic and less diverse. In this case, it seems that these values represent a very specific marine science community – marine biologists. We are not saying that marine biologists are at any fault here, but rather pointing out how specific ways of knowing and valuing the Ocean evolved to dominate the Ocean space. We use Massimi’s (2022, pp. 11) concept of *epistemic severing*, which is “the almost surgical excision of the contribution of particular communities (either within the same scientific perspective or across culturally diverse perspectives) from narratives about scientific knowledge production.” By using this concept, we make an important claim: the report findings are not a result of study limitations, but rather of specific, often historical and structural, events that lead to a dominance of certain forms of knowledge over others. In this case, as Ranganathan (2019) described, the dominant way of knowing and therefore valuing the Ocean is in the context of extraction. How can this be challenged? If extraction of minerals, genetic material, or other materials of economic interest is the main way of valuing the Ocean, specifically the Pacific Ocean, what can we learn when we challenge extraction as the dominant value?

We suggest looking at the challenge as an attempt to recalibrate policy-informing instruments through PNS. How would one “recalibrate” such instruments (e.g., reports)? How can policy-informing science and expertise be more attentive to, and more able to challenge hegemonic practices? The report study area is in the Pacific Ocean. Recall that the Pacific Ocean is, first and foremost, *home* – “a space of interconnection, dialogue, and dynamic relationships between humans, islands, sea, and biota” ( Bode & Yarina, 2020, pp. 78). Angelo Villagomez and Steven Mana’oakamai Johnson (2024) emphasize the Oceanic identity:

*The resurgence of traditional voyaging facilitates a rediscovery of our identities through connection with these thriving ecosystems. All aspects of our unique ways of life are derived from the ocean and the land, including art, song, chants, dance, carvings, religion, myths, stories, architecture, and even economies, world views, and governing systems.*

We emphasize that in these ways of knowing and valuing, the Ocean is not split into disconnected compartments, but is a connected and fluid space. That is, the Ocean is not just a deep seabed with an unrelated water column, as defined and divided by the law. Valuing the Ocean is about appreciating it as a whole and critically appraising divisions imposed on its components and limits through economic valuation. We therefore juxtapose *extraction* and *home* as two distinct ways of knowing and valuing the Pacific Ocean and the Area. This is one way of challenging the extractivist hegemonic way of knowing. The value of the Ocean among Pacific Islanders could be seen as the metaphorical recalibration. We turn back to Ranganathan (2024, pp. 91) and her revealing description of the “burden of translation” that people from Oceania face when trying to engage with ISA – the burden “ *of making their histories and epistemologies comprehensible.”* Most importantly, what we have shown so far is a burden of *epistemic repair* in parallel to *epistemic severing*.

Since the 90s, voices from the Pacific have been building a rich knowledge base of the local Ocean identity – the cornerstone of the value of the Ocean in the region. For lack of a better measure, we look at the 2577 citations (citation total at the time of writing this essay) of Epeli Hau’ofa’s “Our sea of islands.” ^3^ We calculate that the first 20 results (of 2577 citations) on Google Scholar were cited a total of 2,506,487 times. This is a quantitative take on how far the work of Epeli Hau’ofa has travelled. That is, we suggest that many voices have worked over the years to *translate* the value of the Ocean for the people in Oceania. And, perhaps, it also shows that the *burden of translation* in some discussion spaces is already dealt with. What is left is *the burden of epistemic repair*. For that, “Our sea of islands” is an example that shows the value of the Ocean ( Hau’ofa, 1994):

*Oceania is vast, Oceania is expanding, Oceania is hospitable and generous, Oceania is humanity rising from the depths of brine and regions of fire deeper still, Oceania is us. We are the sea, we are the ocean, we must wake up to this ancient truth and together use it to overturn all hegemonic views that aim ultimately to confine us again, physically and psychologically, in the tiny spaces that we have resisted accepting as our sole appointed places, and from which we have recently liberated ourselves. We must not allow anyone to belittle us again, and take away our freedom.*

Even if one does not appreciate valuation studies, and economic valuation in particular, one can still see how Ocean identity is part of the “cultural” services. More specifically, it could fall under “oral traditions and cosmologies” ( DOSI, 2023). And yet, the report did not capture such services. The final output is summarized in Table 2 of the report ( Brander & Guisado Goñi, 2023, pp. 11), which is based on literature and expert consultation ( Brander & Guisado Goñi, 2023, pp. 11). In Table 1, we focus on a snippet of this table from the report, which describes the cultural ecosystem services ( Brander & Guisado Goñi, 2023, pp. 13–14). The categories do not mention oral traditions, cosmologies, or traditional knowledge of the sea and have no description for something that would capture the Ocean identity of the people of Oceania – the Sea of Islands.

**Table 1.** A list of the cultural ecosystem services (from Brander & Guisado Goñi, 2023, pp. 13–14).

	Abyssal plains (nodules)	Seamounts (crusts)	Hydrothermal vents (sulphides)
Existence and bequest value (biodiversity)	Abyssal plains provide habitat for a diverse range of species, including many that are not found anywhere else on Earth. The number, diversity and characteristics of species in the abyssal plain is largely unknown. People may place a value on the continued existence of this unique biodiversity and its conservation for future generations.	Seamounts are biodiversity hotspots that provide habitat for many species of fish, invertebrates, and corals. People may place a value on the continued existence of this unique biodiversity and its conservation for future generations.	Hydrothermal vents are home to unique and specialized species that are adapted to the extreme conditions found in these environments. People may place a value on the continued existence of this unique biodiversity and its conservation for future generations.
Aesthetic	The aesthetic nature of abyssal plains, although only indirectly accessible to the public through media, is of high value for those who appreciate the mysterious and awe-inspiring wonders of the deep ocean.	Seamounts host diverse and vibrant ecosystems and topographical magnificence, provide significant aesthetic value for those who are intrigued by the beauty and complexity of the marine environment.	Hydrothermal vents possess distinct aesthetic qualities due to their unique and specialized biological communities that have adapted to thrive in the extreme and dynamic conditions of the vent environments
Tourism and recreation	Abyssal plains are not accessible for tourism and recreation due to their depth and remote location.	The tourism and recreation service of seamount ecosystems in the Area is limited due to their remote and extreme location.	The tourism and recreation service of hydrothermal vents ecosystems is limited due to their remote and extreme location, as well as their fragility.
Historical archive	Abyssal plains have the potential to serve as important archives of Earth's history, as sediment cores can provide information about past climates and environmental conditions.	Seamounts have the potential to serve as important archives of Earth's history, as they are often associated with past tectonic activity and can provide information about the geological history of the seafloor.	Hydrothermal vents ecosystems provide a valuable historical archive of the Earth's geologic and biological evolution. The minerals and chemical compounds deposited around hydrothermal vents preserve evidence of past geologic events and the evolution of life on Earth. Scientific study of these archives can provide valuable insights into the history and functioning of our planet.

This is a major shortcoming considering the vast knowledge base that Pacific Islanders have generated over millennia of intimate connection with the Ocean. As Mulalap *et al.* (2020) have documented, this includes knowledge about the connectivity of species and marine processes far beyond areas of national jurisdiction, environmental management practices, and a way of knowing the Ocean tied to traditional instrument-free navigation practices within and between coastal communities. A brief list of such traditional knowledge includes knowledge about the behavior of highly migratory species, well-adapted rules for when and how to access and exploit marine resources, and detailed knowledge about Ocean currents and swell patterns. We urge that one should not omit such knowledge based on preconceived ideas about relevance.

It seems that all the history can be forgotten in a valuation report because the legal (UNCLOS definition of the “international seabed”) and institutional arrangements (the Secretariat defines the themes and objectives of the commissioned reports) allow it. The colonial history is seemingly forgotten and the self-determination among Oceanians is forgotten because the Secretariat at ISA relies on specific roles and limits of science to inform policy discussions. The Pacific Ocean becomes a tabula rasa that can be filled with meanings through scientific expertise from outside. Indigenous Islanders are required to enter DSM discussions as if they are marginal knowledge holders or a cultural novelty at the ISA meetings. Yet, in reality, they are central knowledge holders; they embody the Pacific Ocean. While the experts of valuation are experts of literature and expert consultations, the Indigenous Pacific Islanders are experts of their lived reality in Oceania, in the Sea of Islands.

Turning back to the extended peer community, many voices teach us how to increase the quality of policy discussions in the Pacific Ocean (e.g., Fache *et al.*, 2022; Hau‘ofa, 1998; Villagomez & Johnson, 2024). Similarly, discussions exist about how to challenge and disrupt epistemic injustices. Exploring extractivist epistemologies, Alcoff (2022, pp. 16) suggests:

*…four corrective epistemic norms that can counteract extractivist epistemologies: (i) acknowledging the incompleteness of all knowledge, (ii) developing an approach that recognizes plural epistemologies and seeks productive relationships of inter-epistemology; (iii) practicing relational epistemic humility, and (iv) regularizing the assessment of epistemic relationships in projects of knowing.*

Alternatively, thinking about marine science, Massimi (2024) suggests the following remediation:

*Only by recognising the important role and value of local knowledges in scientific knowledge production (marine scientific research in this case), and taking as center-stage the standpoint of multiethnic working-classes (the subaltern), can epistemic severing and epistemic trademarking be averted.*

We want to highlight *epistemic severing* from the above quote. To reiterate, for Massimi (2022, pp. 11) such severing is the intentional or unintentional deletion of various *knowledges*. In the Pacific Ocean, epistemic severing has a long history of knowledge and people exclusion, tokenization and exploitation ( Hau‘ofa, 1994; Hau‘ofa, 1998; Ranganathan, 2019; Villagomez & Johnson, 2024). Hau’ofa (1998, pp. 346–347) once warned that “if we [Pacific Islanders] do not exist for others, then we could in fact be dispensable.” He pointed out that capitalist exploitation in the form of phosphate mining and the Cold War arms race in the form of nuclear testing had already shown that local communities were dispensable in the eyes of the various colonial administrations that dominated the region in the twentieth century. Here, we suggest that the critical negative impacts of the epistemic severing of traditional knowledge of local communities in the ISA Secretariat's report represent a new way of making Pacific Islanders dispensable. It is important to note that the concept of severing, as we see and use it, does not assign blame, it rather invites participants (especially the knowledge makers) to seek repair.

Next, we would like to go back to the question: What does it mean to find ecosystem services that are “key” and of “high economic value”? The above discussion was focused on *finding*. Here, we would like the reader to turn their attention to the word “key. *”* What does it mean to present values as competing and to position some as *key* while leaving others as potentially low-key or missing, as shown above? Does this mean we can make lists of values with competing weights? Assigning a monetary value is heavily criticized in the literature, and the report authors acknowledge such criticism ( Brander & Guisado Goñi, 2023). It is interesting to note that while the authors do not assign a monetary value as such (due to study limitations), they do seem to follow the monetary logic that some ecosystem services have more value than others. Who chooses to assign weight? Can a report improve its quality by acknowledging the alternative choices and refusing to weigh the values? Would policymakers be better informed by acknowledging alternative choices or the biases of experts chosen to assign the weight of values? We leave these questions open for future discussions.

To recap, in this section, we looked at the ecosystem services valuation report commissioned by the ISA’s Secretariat. The Secretariat defines the objectives of the report and selects the consultants and the reviewers for the report. We argue that the final output is one of many examples of knowing and valuing the Area in the Pacific Ocean in extractivist terms above all else. We challenge this extractivist way of knowing and seeing by highlighting some of the works and voices who value it first and foremost as *home* – a stark contrast to extraction. We showed the value of PNS and turned to the extended peer community inside and outside of academia to show how to “recalibrate” the policy instruments that ISA’s Secretariat uses to inform policy discussions at the ISA.

## Final reflections

Decisions about the international seabed cannot always be improved with greater scientific expertise. Therefore, informing the ISA about *the Area* should not be put on the shoulders of scientists alone. PNS shows that more amicable and productive dialogue with civil society should be seen as equally important in the context of policy-shaping. This also gives meaning to the aspirations inscribed in the UNCLOS text. We used PNS and turned to the extended peer community to question the ways of valuing the Area in the Pacific Ocean. We used Massimi’s (2022) concept of *epistemic severing* and suggested *epistemic repair* as lenses to challenge the extractivist image of the Ocean. We aimed to show how one would metaphorically recalibrate policy instruments – expertise and expert outputs that inform policy discussions at the ISA.

To conclude, using PNS as a *knowledge ecosystem-based management* can allow for a consolidated approach to transdisciplinary policy- and decision-shaping. Just like frameworks for Ocean management and governance such as ecosystem-based management, frameworks for knowledge ecosystems allow for the structure of otherwise messy relations. In the policy context, where crucial decisions are shaped by the ISA’s Secretariat, aiming to engage with diverse knowledge communities should increase the quality of the outputs. PNS could improve the public perception of the Secretariat’s work, nurture trust between interested parties, and allow public discourse of the deep seabed to become less contentious. As the newly elected administration of the Secretariat gets to work, we hope it will consider implementing this framework – to reconsider polarization as an opportunity for greater participation, improved dialogue, and shared accountability.

## Data Availability

No data are associated with this article.

## Footnotes

^1^Elections for Secretary-General at ISA have recently concluded on the 2 ^nd^ of August 2024, drawing the administration of Mr. Michael Lodge to a close. We congratulate Letitia Carvalho as the newly elected Secretary-General at ISA.

^2^We use the term keeping in mind the discussion surrounding it ( Sud & Sánchez-Ancochea, 2022).

^3^Hau'ofa, E. (2017). Our sea of islands. In *Peoples of the Pacific* (pp. 429–442). Routledge.
