# Supplementary material for: Science at the Secretariat of the International Seabed Authority: post-normal science for recalibration of policy instruments
Source: Open Res Eur. 2025 Jun 30;4:252. Originally published 2024 Nov 22. [Version 3] doi: 10.12688/openreseurope.18879.3 (PMC12219538; doi:10.12688/openreseurope.18879.3)
Supplement: Peer review history

# Reviewer report: Reviewer response for version 3

**DOI:** 10.21956/openreseurope.22444.r56272

**Published:** 2025-08-21

**Authors:** Elisabetta Menini

**Affiliations:** 1 Duke University, Beaufort, USA

Thank you for considering my previous comments. I believe that most of them have been addressed and clarified where feasible. I still hold some reservations regarding the discussion on the hiring of consultants, where I suggested drawing a parallel with other UN organs to see if the practices are similar, and the section on *how PNS could be used at the Secretariat*. This latter section presents a strong critique of extractivist knowledge systems, but at times, the tone may come across as overly pointed. A more measured framing might help ensure the important message reaches a broader audience and is received as constructive rather than confrontational. However, it is clear from the text that these reflect the authors’ interpretation, and as such, I respect their position.

Is the argument persuasive and supported by appropriate evidence?

Partly

Is the work clearly and cogently presented?

Yes

Is the topic of the essay discussed accurately in the context of the current literature?

Partly

Does the essay contribute to the cultural, historical, social understanding of the field?

Partly

Reviewer Expertise:

Deep Sea Mining Management and policy, International Ocean Governance, Marine Spatial Planning, Knowledge exchange, stakeholder engagement, Marine Science and Conservation

I confirm that I have read this submission and believe that I have an appropriate level of expertise to confirm that it is of an acceptable scientific standard, however I have significant reservations, as outlined above.

# Reviewer report: Reviewer response for version 3

**DOI:** 10.21956/openreseurope.22444.r56273

**Published:** 2025-08-14

**Authors:** Alastair Neil Craik

**Affiliations:** 1 University of Waterloo, Waterloo, Ontario, Canada

I am content to approve the paper without reservations.

Is the argument persuasive and supported by appropriate evidence?

Partly

Is the work clearly and cogently presented?

Yes

Is the topic of the essay discussed accurately in the context of the current literature?

Partly

Does the essay contribute to the cultural, historical, social understanding of the field?

Yes

Reviewer Expertise:

I am an international legal academic with expertise in international environmental law and law of the sea, and have engaged in research on deep seabed mining and the ISA. I have familiarity with the PNS literature.

I confirm that I have read this submission and believe that I have an appropriate level of expertise to confirm that it is of an acceptable scientific standard.

# Reviewer report: Reviewer response for version 2

**DOI:** 10.21956/openreseurope.21326.r51617

**Published:** 2025-05-07

**Authors:** Elisabetta Menini

**Affiliations:** 1 Duke University, Beaufort, USA

Thank you for inviting me to review this manuscript, which offers a valuable contribution to ongoing debates around science and policy at the International Seabed Authority (ISA).

 This essay offers an interesting and timely contribution to the literature on deep-seabed mining (DSM) and the decision-making process at the ISA. I welcome the application of a Post-Normal Science (PNS) framework to counteract the epistemic severing of traditional and Indigenous knowledge from ISA decision-making processes. However, several areas would benefit from improvement and deeper engagement with the relevant literature.

**Is the topic of the essay discussed accurately in the context of the current literature?**

 Considering that this paper is about a very timely topic, it will likely have a wider audience. Therefore, the authors need to define Post Normal Science, knowledge ecosystem, and the implications of using this framework much earlier in the introduction. I would also add for clarity a short sentence in the abstract, like the one you have already in the introduction: "Post-normal science centralizes transdisciplinary through an extended peer community of diverse knowledge holders – a knowledge ecosystem.". The plain language summary facilitates these corrections, but I would choose the words with more care, since it is hard in the context of international and multilateral decision-making to talk about "locals". Who would be the locals in the Area?. I think the summary and the introduction needs work to adapt the definition of PNS into the context of the ISA. 

 While I have to admit that it is the first time that I have heard about PNS, I am familiar with the *Knowledge Exchange* framework and its literature. The PNS seems to have a lot of connection to the co-production of knowledge rooted in Science and Technology Studies and introduced by Sheila Jasanoff in the early 2000. **Co-production of knowledge** is a collaborative process where researchers, policymakers, stakeholders, and/or community members work together to produce knowledge that is both scientifically robust and socially relevant. It emphasizes mutual learning, shared power, and integration of different types of expertise—such as scientific, local, and Indigenous knowledge—to address complex real-world problems. So overall, support the call for a more proactive adoption of co-production/PNS approaches at the ISA, regardless of the institutional or academic modality.

 The introduction on the ISA structure, function and role is well done but I agree with Alastair Neil Craik review, when he say that "the paper should acknowledge the structural limitations that the Secretariat and the other organs of the ISA are subject to". While the Secretariat of the ISA can certainly have a policy shaping input, the Council and the Assembly are the organs that make the decisions, and they receive several inputs from Observer Organizations as well as from Scientific advisors that are chosen by the country representatives and are often part of the delegations. 

 However, it seems that the authors are trying to demonstrate that the Secretariat has more power in shaping the knowledge that forms the baseline information, which is communicated through reports directed to the other organs and the public. I would certainly agree with that, but it might need to be stated more plainly. 

 In my view, while the Secretariat’s influence over high-level decisions such as benefit-sharing may be limited (as that topic remains under negotiation), it does wield procedural power in improving equity through inclusive and holistic knowledge-sharing mechanisms.

**Is the work clearly and cogently presented?**

 Yes, the paper is beautifully written and structured. 

**Is the argument persuasive and supported by appropriate evidence?**

 The argument is persuasive and the evidence is appropriate, but I would argue that it needs a second case study to supplement the analysis of the "Report on the value of ecosystem services and natural capital

 of the Area”. This report is a compelling example, but a comparative case, if feasible, could deepen the analysis. Additionally, identifying which office within the Secretariat commissioned or led the report could provide helpful context. The Secretariat has four offices, which have different responsibilities, and contextualizing when the work was done and what else the Secretariat was doing would help to understand why the report was lacking in integrating the traditional and indigenous knowledge of the Pacific Island States.

 Also, was this report linked to a specific meeting or policy process? If the authors have already clarified this in the text, then feel free to disregard, but the scope and intended use of the report may explain some of its limitations.

 A key point is that monetary and extractive values continue to dominate in multilateral settings like the ISA, as they are widely understood and accepted by member states. While I agree that a shift in priorities is necessary, I also question whether the ISA, as a multilateral policy arena, is currently ready to fully embrace such a shift. 

 Another section that needs work is the section discussing consultants hired by the Secretariat. To offer a fair critique, the authors should either:

1. Analyze the Secretariat’s human resources practices, or
2. Compare the ISA's hiring processes to those of other UN bodies.

Simply citing Bosco et al. (2023) is insufficient. It’s worth exploring whether the ISA Secretariat adheres to UN hiring guidelines, which, while imperfect, provide an administrative benchmark. A similar approach should be applied to the analysis of Observer Organizations—parallels with other UN bodies could make the critique more grounded and balanced. 

**Does the essay contribute to the cultural, historical, and social understanding of the field?**

 I think it does. However, its impact could be significantly enhanced if it were more thoroughly contextualized within the current developments at the ISA. For example, the footnotes mention the election of a new Secretary-General, but that was nearly a year ago. Leticia Carvalho officially began her term in January and appears more open to inclusive, knowledge-diverse governance. However, even before her tenure, the ISA had begun implementing practices to diversify input (as mentioned in Pickens et al. 2024, cited earlier in the paper).

 While these changes are not necessarily sufficient, it is important to recognize that institutional evolution in multilateral settings takes time and depends on broader political dynamics. For this reason, a more nuanced critique, showing how different perspectives were considered before reaching the authors' conclusions, would strengthen the paper.

Is the argument persuasive and supported by appropriate evidence?

Partly

Is the work clearly and cogently presented?

Yes

Is the topic of the essay discussed accurately in the context of the current literature?

Partly

Does the essay contribute to the cultural, historical, social understanding of the field?

Partly

Reviewer Expertise:

Deep Sea Mining Management and policy, International Ocean Governance, Marine Spatial Planning, Knowledge exchange, stakeholder engagement, Marine Science and Conservation

I confirm that I have read this submission and believe that I have an appropriate level of expertise to confirm that it is of an acceptable scientific standard, however I have significant reservations, as outlined above.

# Response

**Published:** 2025-06-16

**Authors:** aistė klimašauskaitė

**Affiliations:** University of Bergen, Norway

Dear Elisabetta Menini,

 Thank you for your detailed and thoughtful comments. We appreciate you sharing your professional knowledge of the ISA and hope that the provided answers clarify some of our choices. Note that your comments were shortened, where possible, but the responses address all the issues raised in the comments.

**Comment:** “Who would be the locals in the Area?”

**Response:** We would like to stress that voices from the Pacific are the locals.

**Comment:** “While I have to admit that it is the first time that I have heard about PNS, I am familiar with the Knowledge Exchange framework and its literature. The PNS seems to have a lot of connection to the co-production of knowledge rooted in Science and Technology Studies and introduced by Sheila Jasanoff in the early 2000.”

**Response:** We would like to clarify the differences between PNS, Jasanoff’s idiom of “co-production”, and the literature on knowledge co-production among diverse actors.

 A useful comment on what “co-production” is can be found on Jasanoff’s blog:

 “Much of my work elaborates the notion of co-production: the simultaneous processes through which modern societies form their epistemic and normative understandings of the world. This framework, most systematically laid out in States of Knowledge, shows how scientific ideas and beliefs, and (often) associated technological artifacts, evolve together with the representations, identities, discourses, and institutions that give practical effect and meaning to ideas and objects.”

 The blog was accessed by following: https://sheilajasanoff.org/research/co-production/

 So, for Jasanoff, co-production is not an approach to improve science for policy work as it is for PNS. PNS is different from knowledge co-production as well, being a framework in science for policy space under the specific conditions as noted in our essay:

 “PNS is applicable in situations ‘where facts are uncertain, values in dispute, stakes high and decisions urgent’ (Funtowicz & Ravetz, 2020).”

 We stress, however, what was explained in our paper: the urgency surrounding deep-sea mining is socially constructed by the industry, specifically the deep-sea mining startups.

 By contrast, knowledge co-production is broader and less related to situations that PNS aims to tackle, as the reviewer suggests: “Co-production of knowledge is a collaborative process where researchers, policymakers, stakeholders, and/or community members work together to produce knowledge that is both scientifically robust and socially relevant. It emphasizes mutual learning, shared power, and integration of different types of expertise—such as scientific, local, and Indigenous knowledge—to address complex real-world problems.”

 In sum, the following perspective article clarifies some of the differences and connections among knowledge co-production approaches:

 Norström, A. V., Cvitanovic, C., Löf, M. F., West, S., Wyborn, C., Balvanera, P., ... & Österblom, H. (2020). Principles for knowledge co-production in sustainability research. *Nature sustainability*, *3*(3), 182-190.

**Comment:** “The introduction on the ISA structure, function and role is well done but I agree with Alastair Neil Craik review, when he say that "the paper should acknowledge the structural limitations that the Secretariat and the other organs of the ISA are subject to". (...) In my view, while the Secretariat’s influence over high-level decisions such as benefit-sharing may be limited (as that topic remains under negotiation), it does wield procedural power in improving equity through inclusive and holistic knowledge-sharing mechanisms.”

**Response: **We agree with the reviewer that ISA has various bodies which receive diverse inputs. We do not claim that the Secretariat is the only source of inputs that enter the negotiations. As stated in some of the previous replies to reviewers, choosing a specific level of analysis allows the presentation of the applicability of PNS in one specific context – the work of the Secretariat. This does not mean that PNS is not applicable in other contexts, other bodies at the ISA, or that this choice of a context disputes the existence of other inputs.

 As replied to Dr. Craik, we do not dispute the legal limits of the Secretariat’s work; we simply highlight its practices and the role in commissioning science for informing the negotiations at the ISA. This particular report is part of “Studies, Reports and Workshop Reports” for the Mining Code:

https://www.isa.org.jm/the-mining-code/studies-reports-and-workshop-reports/ 

 In the response to Dr. Craik, we detailed how the Secretariat had a role in the report, including its impact on who the selected experts were to be interviewed for the report. We feel those details are sufficient to show why we chose the Secretariat as a useful example.

 We also would like to highlight footnote number 100 from the Bosco et al. (2023) work, which details the role of the Secretariat, in particular:

 “ *See, e.g*., Dale Squires et al., “Equitable sharing of financial and other economic benefits from deep-seabed mining” (Kingston: International Seabed Authority, 2021) (systematic report on potential benefit-sharing regimes, including recommendations, authored by the Secretary-General with several outside experts and consultants); compare “Development of rules, regulations and procedures on the equitable sharing of financial and other economic benefits derived from activities in the Area pursuant to section 9, paragraph 7 (f), of the annex to the Agreement relating to the Implementation of Part XI of the United Nations Convention on the Law of the Sea of 10 December 1982,” ISBA/26/A/24-ISBA/26/C/39 (2021) (FC’s report on equitable sharing, borrowing heavily and often word for word from the technical study and antecedent Secretariat reports); “Summary report of the Chair of the Legal and Technical Commission on the work of the Commission during the twentieth session of the International Seabed Authority,” ISBA/20/C/20 (2014), para. 23 (noting the work of the Secretary-General and a consultant on the initial exploitation regulations draft); “Draft regulations on exploitation of mineral resources in the Area,” ISBA/23/C/12 (2017), para. 1 (noting the SG prepared the first exploitation regulations draft at the LTC’s request); “Election of members of the Legal and Technical Commission,” ISBA/23/C/2 (2016); “Election of members of the Legal and Technical Commission,” ISBA/26/C/14 (2020) (two of a number of reports the SG has written for the Council about the LTC’s composition). While in any one case the line between facilitating another organ’s work and taking on its responsibilities is hard to pinpoint, the Secretariat’s disproportionate output is striking: on average, from the first to 26th sessions the Secretariat authored about 56% of the ISA’s reports and about half of its official documents overall.”

 In sum, we did not focus on the details about the Secretariat’s impact, as such analyses are already available; our section on the role of the Secretariat is meant to highlight those works and to show the rationale of our choice. In sum, we mainly focus on the value of PNS while using the previous work to select the Secretariat as a relevant body at the ISA.

**Comment:** “The argument is persuasive and the evidence is appropriate, but I would argue that it needs a second case study to supplement the analysis of the "Report on the value of ecosystem services and natural capital

 of the Area”. This report is a compelling example, but a comparative case, if feasible, could deepen the analysis. Additionally, identifying which office within the Secretariat commissioned or led the report could provide helpful context. The Secretariat has four offices, which have different responsibilities, and contextualizing when the work was done and what else the Secretariat was doing would help to understand why the report was lacking in integrating the traditional and indigenous knowledge of the Pacific Island States.”

**Response:** We agree with the reviewer, but also would like to stress that the purpose of our essay is not to seek why the report was lacking something; rather, the purpose is to present PNS as a framework to improve the quality of the commissioned reports overall. We would like to stress that many reports, documents and other texts can be examples to be chosen to apply the PNS framework.

 As detailed in the replies to Dr. Craik’s and as noted in the above response, the report is part of the studies that were commissioned for the Mining Code. As we presented in the previous responses and in the paper itself, the report is an illustrative example. The value of an example is simply to show how PNS brings more quality into the commissioned reports. The example in itself does not state that other reports have or do not have limitations. The example simply shows how commissioned reports can improve their quality. In short, we do not aim “to deepen the analysis” about the work at the Secretariat, as such works already exist (e.g., Bosco et al. 2023); we aim to show the value of PNS.

**Comment:** “A key point is that monetary and extractive values continue to dominate in multilateral settings like the ISA, as they are widely understood and accepted by member states. While I agree that a shift in priorities is necessary, I also question whether the ISA, as a multilateral policy arena, is currently ready to fully embrace such a shift.”

**Response:** We agree with the reviewer and do not expect a shift to happen because this article was written. The purpose of the article is to contribute to an open conversation as to how such a shift might increase the quality of some of the work.

**Comment:** “Another section that needs work is the section discussing consultants hired by the Secretariat. To offer a fair critique, the authors should either:

1. Analyze the Secretariat’s human resources practices, or
2. Compare the ISA's hiring processes to those of other UN bodies.

Simply citing Bosco et al. (2023) is insufficient. It’s worth exploring whether the ISA Secretariat adheres to UN hiring guidelines, which, while imperfect, provide an administrative benchmark. A similar approach should be applied to the analysis of Observer Organizations—parallels with other UN bodies could make the critique more grounded and balanced.”

**Response:** We agree that deeper exploration can give more insights into how the work happens. Yet again, the purpose of our essay is focused on how to improve the quality of work and not so much to analyze how the work currently happens (we use that only as context). As in the replies to Dr. Craik, we did detail how, for example, specific people were involved and how they are connected to the Secretariat and one of the mining contractors (this is in addition to the analysis provided by Bosco et al. 2023). We believe such details are enough to show why a conversation about improving the practices is of value. We also believe that the work of Bosco and colleagues (2023) provides a very detailed account of how the Secretariat works and is one of the reasons why we chose the Secretariat as an example for our essay. In sum, we do not aim to critique; we aim to propose that PNS is a useful framework.

**Comment:** “However, its impact could be significantly enhanced if it were more thoroughly contextualized within the current developments at the ISA. (...) While these changes are not necessarily sufficient, it is important to recognize that institutional evolution in multilateral settings takes time and depends on broader political dynamics. For this reason, a more nuanced critique, showing how different perspectives were considered before reaching the authors' conclusions, would strengthen the paper.”

**Response:** We agree with the reviewer, but would also like to stress that the change of the Secretary should not condition the understanding of how quality science could be produced through the work of the Secretariat. The PNS approach adds value no matter which SG is in charge. The essay also does not deny that ISA has implemented practices to diversify inputs. The essay proposes a framework. To reiterate, we give an analogy of ecosystem-based management, which is widely accepted. We highlight that no similar framework is used for knowledge production within the Secretariat and propose PNS. We use the analogy to show the usefulness of frameworks in general.

 We thank the reviewer again and hope that our answers clarify the purpose of the essay and the arguments presented within it.

 Kind regards,

 The authors

# Reviewer report: Reviewer response for version 2

**DOI:** 10.21956/openreseurope.21326.r51849

**Published:** 2025-03-22

**Authors:** Alastair Neil Craik

**Affiliations:** 1 University of Waterloo, Waterloo, Ontario, Canada

The paper presents an argument that the knowledge ecosystem of the ISA is too tightly drawn, favouring traditional scientific approaches at the expense of other knowledge systems and ways of knowing, The article focuses on the role of the Secretariat in shaping the knowledge environment for policy decisions. The argument draws on the preparation of a single report on ecosystem services as an example of the ISA Secretariat’s approach and to demonstrate how PNS may be used to broaden the knowledge ecosystem.

**Is the topic of the essay discussed accurately in the context of the current literature?**

 The paper draws appropriately on the literature on the ISA but engages in a limited fashion with the scientific literature, particularly the literature describing the extent and nature of scientific uncertainty (citing only one paper Amon et al 2022; see also Levin 2016; Niner et al 2018; Levin, Amon, Lily 2020). Given the centrality of uncertainty to PNS – this point could be better substantiated with references to other papers.

**Is the work clearly and cogently presented?**

 Stylistically – the paper is clear and well-written. The structure is logical and flows well.

 There is little discussion of the broader legal context, which is relevant in the context of this paper. UNCLOS was negotiated in the 1970’s when the prevailing epistemic models were very much rooted in a traditional scientific (western) paradigm. The treaty as a whole reflects this. I appreciate that the intent of the argument is to call for an opening up of this paradigm – but the paper should acknowledge the structural limitations that the organs of the ISA are subject to. The argument that is suggested but not developed – that article 136 can be used to open up the epistemic limitation of the Convention – could be elaborated upon and linked to key policy questions discussed such as benefit sharing.

 Focusing on the Secretariat is sound in light of the points presented on the significant role that the Secretariat plays in managing the knowledge environment. However, the author’s do not acknowledge the legal constraints that the Secretariat operates under. Specifically, the Secretariat is bound by the treaty and must take its direction from states through the Assembly and Council. The question that is not addressed is whether it is solely up to the parties to determine the scope of the knowledge environment (and to widen it from that found in UNCLOS) or whether the S-G can exercise a degree of agency to address perspectives that go beyond the statist and extractivist orientation of Part XI. The literature cited, accurately portrays the degree of independence that the Secretariat acts with but are also critical of the degree of unsupervised authority and acknowledge that key substantive direction must flow from the political bodies.

 In this regard, the criticism at p, 6 is unconvincing.  – The authors criticize Lodge (writing not in an official capacity – so it may be inaccurate to attribute this to the institution of the Secretariat)  for failing to recognize the full context of Indigenous benefit sharing. Specifically, the criticism notes the failure to acknowledge that the ocean is “home” to local people. The concept of the ocean as “home” is, however, complex and contested, as is the basis upon which benefits may be conferred. There is a lack of clarity, within UNCLOS and international law generally, including the UNDRIP, as to the basis of benefit sharing with Indigenous people – a point acknowledged in the paper cited. For example, the benefit sharing provision of UNCLOS (art 140) are principally directed at inter-state benefit sharing from Activities in the Area. The critique of failing to acknowledge Indigenous perspectives in the benefit sharing debate is an artefact of UNCLOS, and less a determination of the Secretariat.

**Is the argument persuasive and supported by appropriate evidence?**

 The focus on the single report provides limited substantiation of the overall argument. First, at p 5 the authors note that PNS does not replace scientific reports but rather is a method for informing policy. The report acknowledges that the perspective provided does not represent the full spectrum of views. The suggestion seems to be that these perspectives ought to be reflected in each policy input, as opposed to being reflected in the broader policy process (the “ecosystem”). In many policy processes, different ways of knowing are brought into a policy process through distinct reports, often prepared by Indigenous knowledge keepers or with their collaboration, through traditional knowledge studies. It is not clear whether the authors are arguing that each input ought to engage with a variety of epistemologies, (which the report authors may not be trained for). In the deep seabed mining context, there may be many instances where specific knowledge is required. For example, plume dispersal studies of mining activities, which require specific expertise. This knowledge may inform a broader policy question concerning the determination of serious harm – but this policy question may also be informed by other perspectives including the social and cultural harm that mining activities may cause to local and Indigenous peoples – but projected into the policy process through a different input. In other words “epistemic repair” may take a number of forms, and the appropriate form may be contextually determined. In the case of the ecosystem services report, we do not know (from the paper) what policy process this report is intended to inform or what other input may be anticipated. It is hated to assess, whether the epistemic limitation of that report were (or ought to be) addressed elsewhere.

 There may also be an attribution problem here in that it is not clear the degree to which the substance of the report is the result of the Secretariat’s direction.

**Does the essay contribute to the cultural, historical, social understanding of the field?**

 The gist of the argument – that the ISA would benefit from an approach to knowledge ecosystem management that accounts for the full range of relevant knowledges and ways of knowing – is a useful contribution and situating that argument in PNS is especially useful because it attaches the argument to two critical characteristics of the DSM regime: scientific uncertainty and values plurality. The metaphor is an apt one – and may resonate with the ISA policy community that has familiarity with the broader concept of ecosystem management.

 The prescriptive recommendation (at p.11) is for the Secretariat to adopt a framework rooted in PNS, but the contours of the framework are unclear. The study focuses on one element of the “ecosystem” but does not identify or address the other elements of the ecosystem. It would, in my view, be very difficult for the Secretariat or political organs of the ISA to action this recommendation without more details.

 In summary the paper, makes a valuable contribution to the scholarly and policy debates on the approach to knowledge management that would be appropriate for the DSM context. However, the paper needs to acknowledge and address the two principal limitations discussed above.

Is the argument persuasive and supported by appropriate evidence?

Partly

Is the work clearly and cogently presented?

Yes

Is the topic of the essay discussed accurately in the context of the current literature?

Partly

Does the essay contribute to the cultural, historical, social understanding of the field?

Yes

Reviewer Expertise:

I am an international legal academic with expertise in international environmental law and law of the sea, and have engaged in research on deep seabed mining and the ISA. I have familiarity with the PNS literature.

I confirm that I have read this submission and believe that I have an appropriate level of expertise to confirm that it is of an acceptable scientific standard, however I have significant reservations, as outlined above.

# Response

**Published:** 2025-06-16

**Authors:** aistė klimašauskaitė

**Affiliations:** University of Bergen, Norway

Dr. Alastair Neil Craik,

 Thank you for the detailed and thoughtful comments. We have listed all the responses below and will update the manuscript accordingly.

**Comment:** “The paper draws appropriately on the literature on the ISA but engages in a limited fashion with the scientific literature, particularly the literature describing the extent and nature of scientific uncertainty (citing only one paper Amon et al 2022; see also Levin 2016; Niner et al 2018; Levin, Amon, Lily 2020). Given the centrality of uncertainty to PNS – this point could be better substantiated with references to other papers.”

**Response: **It is an important point and the suggested works are highly valuable in the discussion. We will update the manuscript and include the papers that touch on scientific uncertainty and its role in regulations and management:

 Ginzky, H., Singh, P. A., & Markus, T. (2020). Strengthening the International Seabed Authority's knowledge-base: addressing uncertainties to enhance decision-making. *Marine Policy*, *114*, 103823.

 Haeckel, M., Vink, A., Janssen, F., & Kasten, S. (2020). Environmental impacts of deep seabed mining. In *New knowledge and changing circumstances in the law of the Sea* (pp. 327-340). Brill Nijhoff.

 Roest, W. R., Clark, M. R., & Brekke, H. (2023). The scientific challenges of deep-sea mining. In *Routledge handbook of seabed mining and the law of the sea* (pp. 11-32). Routledge.

 We will also improve the use of the cited literature (Blanchard et al., 2023; Bosco et al., 2023; Pickens et al., 2024; Wilde et al., 2023) that highlights the outstanding regulatory challenges and uncertainties (to highlight the regulatory uncertainty in addition to the scientific uncertainty).

**Comment: “**There is little discussion of the broader legal context, which is relevant in the context of this paper. UNCLOS was negotiated in the 1970’s when the prevailing epistemic models were very much rooted in a traditional scientific (western) paradigm. The treaty as a whole reflects this. I appreciate that the intent of the argument is to call for an opening up of this paradigm – but the paper should acknowledge the structural limitations that the organs of the ISA are subject to. (...)”

**Response:** These are important points, and we partially acknowledge the historical context (e.g., works of Ranganathan, Massimi). We also stress the contradictory mandate of mining and protection in the second paragraph of the essay (Part XI talks about “protection and preservation” in addition to mining). While UNCLOS does impose legal constraints, many of those constraints are broad, and it is up to the negotiations among the delegations to decide, for example, what goes into the Mining Code. We focus only on the role of the Secretariat, which, through a variety of science and expert reports, informs the Mining Code developments, where the ecosystem valuation report (Brander & Guisado Goñi, 2023) appears as well:

 https://www.isa.org.jm/the-mining-code/studies-reports-and-workshop-reports/

 Moreover, science and scientific methodologies progress with time and are not restricted by the UNCLOS text, as we acknowledge in our essay.

 We agree on the key role of the delegations. But at the same time, we also acknowledge the current role of the ISA’s Secretariat and propose PNS in the current role and capacity of the Secretariat.

**Comment:** “In this regard, the criticism at p, 6 is unconvincing. (...) The critique of failing to acknowledge Indigenous perspectives in the benefit sharing debate is an artefact of UNCLOS, and less a determination of the Secretariat.”

**Response:** We note that the paper by Mr. Lodge and colleagues does state the following: “The views expressed in this article are those of the authors, and do not necessarily reflect the position of the International Seabed Authority or any of its member State.”

 Yet, it is important to note that the affiliation of Mr. Lodge is also stated as: “Secretary-General, International Seabed Authority, Kingston, Jamaica”

 Overall, it is not easy to detangle the official roles and the views.

 Next, the paper by Lodge and colleagues describes some of the legal challenges. Still, the dual role of ISA as mandated by UNCLOS is to exploit and to protect, something that Mr Lodge acknowledges in the title of their paper: “Sharing and Preserving the Resources in the Deep Sea.” So, describing non-monetary values seems not out of place, bearing in mind that delegations at ISA include delegates from the Pacific Islands.

 We argue that it is not contradictory for the SG to consider the meanings of the seabed in the suggested context of “home.” That is, if the role of ISA is to protect and exploit, then it is not contradictory to discuss monetary benefits together with non-monetary benefits (money vs home), as the paper by Lodge and colleagues discusses ecosystem services too. Ecosystem services are tightly connected to the acknowledgement of home, for example, Professor Sir Partha Dasgupta calls nature “our home” (https://www.cam.ac.uk/stories/dasguptareview). In addition, we cite Epeli Hau’ofa (1994) to show the longstanding discussion about the Pacific Islanders and their “Sea of Islands.”

**Comment:** “The focus on the single report provides limited substantiation of the overall argument. (...) It is hated to assess, whether the epistemic limitation of that report were (or ought to be) addressed elsewhere. There may also be an attribution problem here in that it is not clear the degree to which the substance of the report is the result of the Secretariat’s direction.”

**Response:** We agree with the reviewer. While the broader idea is to normalize the PNS approach at ISA, it was easier to explore PNS role in the context of one specific ISA body. Narrower context allows more clarity.

 The local community in the Pacific is an integral rights-holding group that should be visible in relevant documents and discussions. So, ideally, it would not be “projected into the policy process through a different input” as the reviewer suggests. The very simple reason for it is that such separation potentially creates artificial separations in the policy debate and allows it to, in part, distort reality.

 It is indeed often hard to know how the information plays out in the negotiations, and unless such reports are openly quoted in the publicly available information about the negotiations at ISA, it can be challenging to assess their role. Based on the documents available to us, we can see that the report was mentioned by one of the ISA’s observers at the “Third part of the 28th session of the International Seabed Authority”:

 https://www.dosi-project.org/wp-content/uploads/Participation-report-28th-Session-ISA.pdf

 The mention appears as follows: “Third, DOSI suggests that the effects on ecosystem services are assessed as part of the EIA, and we take the opportunity to remind the council that there is a lack of data to estimate the cost to ecosystem services in the deep sea, as highlighted by a report from the ISA (ISA/2023/OEMMR002). This work ought to be a high research priority.”

 Therefore, we see it (the report) as having a role in the negotiations, and the quote indicates support for the need for similar work.

 We want to stress that the report is listed among the “Studies, Reports and Workshop Reports” that inform the Mining Code:

https://www.isa.org.jm/the-mining-code/studies-reports-and-workshop-reports/

 So, in itself, this report should be seen as one of the key reports informing the Mining Code negotiations. Yet, we do agree with the reviewer that other inputs might be anticipated in addition to this report, even though the above link does not list any other ecosystem valuation studies. Still, we see the report as a very valuable example of epistemic omissions and believe that the value of the essay is not diminished.

 While we cannot provide the full information on “the degree to which the substance of the report is the result of the Secretariat’s direction”, we would like to clarify that the overall role of the Secretariat in hiring outside consultants’ work lacks transparency (Bosco et al., 2023, pp. 17). Since one of the reviewers for the report is Dr. Dale Squires (mentioned in the acknowledgements section on the pp. 35 of the report (Brander & Guisado Goñi, 2023)) who is the co-author of at least two papers led by Mr. Lodge.

 It is worth noting that the other reviewer of the report (Brander & Guisado Goñi, 2023, pp. 35) is Dr. Saleem Ali, who occasionally appeared in the context of ISA work in various capacities. For example, he appeared as an expert in another Secretariat’s report:

 https://www.isa.org.jm/news/newly-released-independent-report-highlights-contributions-international-seabed-authority/

 Moreover, while the report is labelled as “independent”, the foreword by Mr. Lodge in the report (pp. 5) clearly states the role of the Secretariat in selecting experts for interviews to inform the report by saying “we deliberately chose”:

 “I also wish to thank all those who volunteered to be interviewed for the purposes of the report, whose names are listed in Annex 1. In selecting potential interviewees, we deliberately chose as wide a range of views as possible, and I believe this is reflected in the report.”

 In other words, the “we” indicates that the Secretariat not only had an administrative role as noted in the document’s acknowledgements (page 2), but also a significant role in impacting the content of the report. Moreover, on page 48, where the reader finds the list of experts that were interviewed for the report, the following is stated:

 “People marked with asterisks were interviewed in their personal capacity as members of the ISA Secretary General’s Experts Group on the Sustainable Development Goals, and not in their official capacity.”

 Dr. Saleem Ali is marked with an asterisk. Note that six interview participants out of 27 appear to be part of the SG’s expert group (described on pp. 49 of the report  (Brander & Guisado Goñi, 2023)). While this is not something we plan to use in our essay, such information provides insights into the roles of the Secretariat and their commissioned work.

 Finally, it is important to note that besides being an expert for the SG’s expert group, Dr. Saleem Ali is also presented at the Metals Company (this is one of the mining contractors at the ISA through its subsidiary Nauru Ocean Resources Inc., former DeepGreen) website (https://metals.co/timeline/) as part of their Science Advisory Board. Dr. Ali published works that were sponsored by this deep-sea mining company. In sum, these details show the role of the Secretariat and the SG.

**Comment:** “The gist of the argument – that the ISA would benefit from an approach to knowledge ecosystem management that accounts for the full range of relevant knowledges and ways of knowing – is a useful contribution and situating that argument in PNS is especially useful because it attaches the argument to two critical characteristics of the DSM regime: scientific uncertainty and values plurality. (...) The prescriptive recommendation (at p.11) is for the Secretariat to adopt a framework rooted in PNS, but the contours of the framework are unclear. (...)”

**Response:** We will update the new version to highlight the key practice work from a special issue focused on PNS practices:

 Dankel, D. J., Vaage, N. S., & van der Sluijs, J. P. (2017). Post-normal science in practice. *Futures*, *91*, 1-4.

 While there are several approaches as described in the special issue, we will highlight the following:

 Pereira, Â. G., & Saltelli, A. (2017). Post-normal institutional identities: Quality assurance, reflexivity and ethos of care. *Futures*, *91*, 53-61.

 Pereira and Saltelli (2017) summarize PNS practice as: “...a commitment to three intertwined and complementary cultures, which embrace the post-normal science framework: that of quality assurance by an extended peer community: that of reflexivity and critical thinking; and finally that of an ethos of care.”

 Thank you again for the detailed and helpful review comments. We hope that our answers clarify some of the choices and arguments made in the essay.

 Kind regards,

 The authors

# Reviewer report: Reviewer response for version 2

**DOI:** 10.21956/openreseurope.21326.r51573

**Published:** 2025-03-07

**Authors:** Matteo De Donà

**Affiliations:** 1 Lund University, Lund, Sweden

Dear Authors,

 Thank you for the detailed answers you provided to my comments. I believe you have done a commendable job in addressing my major concerns and provided sufficient clarifications/additions to the manuscript.

 My only remaining concern relates to the ‘metaphorical’ part of your proposed ‘recalibration’. Neither the manuscript text nor the response to my comment really clarify what this metaphor is about. I honestly fail to see any metaphor there (if any, the only figure of speech I detect is a simile between ecosystem-based management and knowledge ecosystem-based management). I therefore do not see the need for adding the word ‘metaphorical’ to ‘recalibration’. I recommended that you either 1) delete references to ‘metaphorical’ or 2) thoroughly explain why recalibration has to be ‘metaphorical’.

 I wish you best of luck with the finalization of the manuscript.

 Kind regards,

 Matteo De Donà

Is the argument persuasive and supported by appropriate evidence?

Partly

Is the work clearly and cogently presented?

Partly

Is the topic of the essay discussed accurately in the context of the current literature?

Yes

Does the essay contribute to the cultural, historical, social understanding of the field?

Yes

Reviewer Expertise:

Global environmental governance, international relations (IR), Science and technology studies (STS)

I confirm that I have read this submission and believe that I have an appropriate level of expertise to confirm that it is of an acceptable scientific standard.

# Reviewer report: Reviewer response for version 1

**DOI:** 10.21956/openreseurope.20429.r47319

**Published:** 2024-12-17

**Authors:** Matteo De Donà

**Affiliations:** 1 Lund University, Lund, Sweden

Thank you for the opportunity to read this informative and timely manuscript on the role of knowledge in informing policy-making in the framework of a relatively under-researched international organization, namely the International Seabed Authority (ISA).

 Based on the analysis of existing literature and relevant policy documents, the authors investigate the practices of knowledge-making within the ISA, discussing the deeper implications of such practices. Arguing that the agency and influential role of the ISA Secretariat in framing marine science and knowledge has led to problematic patterns of ‘epistemic severing’, the authors attempt to identify a remediation (or ‘epistemic repair’) for what they perceive as an instance of epistemic justice in intergovernmental environmental policy. In this regard, the authors advocate the adoption of a ‘knowledge ecosystem-based management’ approach based on the tenets of post-normal science (PNS). According to the authors, such an approach would have the potential to enhance the legitimacy of the ISA Secretariat and to foster a climate of trust and dialogue among ISA stakeholders.

 I believe that the manuscript represents a valuable contribution to knowledge on the contested status of science and expertise in intergovernmental politics. In this respect, the authors point to important research and policy gaps, offering promising avenues for imagining and assessing policy-relevant knowledge in international environmental governance. Nevertheless, there is significant room to improve the manuscript in terms of form and content. In particular, while some concepts and statements could be presented with enhanced clarity and accuracy, the key arguments advanced in the paper should be more convincingly and concretely articulated. I provide below more detailed comments and recommendations: 

  

1. First of all, it would be beneficial to provide the reader with a better understanding of the relevance and meaning of the case study at hand. I suppose that a clarification of the scholarly audience to which this manuscript is addressed may facilitate the authors’ task. The impression is that the gist of the paper is revolving around epistemic injustice. If the latter is the core focus of the paper, this should be emphasized from the very beginning, including a brief account of how this question has been dealt with in other relevant instances of science–policy interaction
2. Related to the above point, it would be helpful to understand how unique is the case of the ISA. The authors dedicate substantial space to describe the conspicuous role played by the Secretariat in orchestrating the activities of the ISA. However, this is may not be new to many scholars and practitioners, as the tendency of international secretariats to ‘pull the strings’ of various regimes is well-documented (for instance, in the global governance literature). Moreover, the three conditions detailed by Funtowicz and Ravetz (2020) are not unique to the ISA but likely to emerge in several other international environmental policy areas. I would therefore invite the authors to better account for why the ISA is a case that deserve scholarly and policy attention. Doing so would also help determine whether the authors’ policy recommendations about the potential of PNS could be applicable to other cases of international organizations or multilateral environmental agreements
3. Several notions and concepts are mentioned in the manuscript. However, in some cases, no or limited explanation is provided. It would be important to briefly elucidate the meaning of these concepts for readers who are not familiar with the context (e.g. ‘blue growth wave’). In addition to the key concepts that will be discussed in the next points, I believe that especially two notions deserve clarification: a) ‘the Area’. The meaning of this notion is not straightforward and a better explanation of it should be provided at the very beginning of the paper; b) ‘transformative actions’. This expression is mentioned in central passages of the manuscript to indicate a key end goal: can the authors explain what they mean by it?
4. I would invite the authors to reflect more in-depth on the nature of ‘epistemic severing’, as this concept is central to the author’s argument. Do the authors understand epistemic severing as a deliberate or inadvertent practice? Clarifying this would be particularly important also in light of the strong claims made on page 9 (“Here, we suggest that the critical negative impacts of the epistemic severing of traditional knowledge of local communities in the ISA Secretariat’s report represent a new way of making Pacific Islanders dispensable”).
5. It is unclear what “metaphorically recalibrate” exactly means. Does it mean that the contribution of PNS would be limited to a cosmetic discursive shift? I suspect that the authors’ ambition is higher than that. If so, can the authors describe in more detail how PNS could foster a knowledge ecosystem in practice? In other words, what should the Secretariat do to operationalize the ideal of PNS and translate it into action? The example presented by the authors is useful in showing what the ISA Secretariat could do as far as the content of its policy documents is concerned (i.e. broadening the knowledge base of the reports and ensuring room for alternative epistemic framings therein). However, the questions remain: how could the Secretariat go about ‘epistemic repair’ in practice? Is enriching reports with inputs from various knowledges sufficient to this end? I suspect that the answers may lie in a more detailed articulation of what a ‘knowledge ecosystem-based management’ would consist of. At the moment, no such account is provided in the manuscript. Offering more clarity on this aspect may substantially increase the policy-relevance of the paper, especially if one of its aims is informing the work programme to be undertaken by the upcoming ISA Secretary-General
6. Besides the purported benefits of PNS (“improve the public perception of the Secretariat’s work, nurture trust between interested parties, and allow public discourse on the deep seabed to become less contentious”), have the authors considered the possible challenges that the Secretariat may encounter in implementing their proposed approach?
7. Language & style. There a few grammar imperfections, including redundancies. The syntax should also be improved, as some sentences are ill-constructed or unnecessarily convoluted. Before acceptance, I recommend that the manuscript be thoroughly proofread. 

Is the argument persuasive and supported by appropriate evidence?

Partly

Is the work clearly and cogently presented?

Partly

Is the topic of the essay discussed accurately in the context of the current literature?

Yes

Does the essay contribute to the cultural, historical, social understanding of the field?

Yes

Reviewer Expertise:

Global environmental governance, international relations (IR), Science and technology studies (STS)

I confirm that I have read this submission and believe that I have an appropriate level of expertise to confirm that it is of an acceptable scientific standard, however I have significant reservations, as outlined above.

# Response

**Published:** 2025-01-21

**Authors:** aistė klimašauskaitė

**Affiliations:** University of Bergen, Norway

Dear Dr. De Donà,  

 Thank you for the comments. Below, we provide answers to all the comments. We will upload an updated manuscript to the ORE platform.    

**Question:**

 1. First of all, it would be beneficial to provide the reader with a better understanding of the relevance and meaning of the case study at hand. I suppose that a clarification of the scholarly audience to which this manuscript is addressed may facilitate the authors’ task. The impression is that the gist of the paper is revolving around epistemic injustice. If the latter is the core focus of the paper, this should be emphasized from the very beginning, including a brief account of how this question has been dealt with in other relevant instances of science–policy interaction

**Answer:** This is an important comment, it shows us that we need to emphasize the core thesis of the text – the value of PNS in policy advice space. We would like to clarify that this is not a case study, but rather an essay focused on PNS with an illustrative example of one valuation report. The example that we chose regarding ecosystem valuation is just one example of all the possible uses of the PNS framework. The core message of the paper is PNS and its key value – the value of knowledge diversity in policy advice context, which depends on a specific context/example at hand and is not necessarily about epistemic justice. These aspects are highlighted in the abstract and the introduction.  

 As we show below, in some cases of intergovernmental policy space such as at ICES, IPBES and IPCC, there has been attention to the question of knowledge in the policy context. We will update the manuscript to show these relevant examples.  

 To clarify the above, we will add a paragraph at the end of the introduction section, where we will repeat the main argument and present the sections of the essay in the context of the argument.  

**Question:**

 2. Related to the above point, it would be helpful to understand how unique is the case of the ISA. The authors dedicate substantial space to describe the conspicuous role played by the Secretariat in orchestrating the activities of the ISA. However, this is may not be new to many scholars and practitioners, as the tendency of international secretariats to ‘pull the strings’ of various regimes is well-documented (for instance, in the global governance literature). Moreover, the three conditions detailed by Funtowicz and Ravetz (2020) are not unique to the ISA but likely to emerge in several other international environmental policy areas. I would therefore invite the authors to better account for why the ISA is a case that deserve scholarly and policy attention. Doing so would also help determine whether the authors’ policy recommendations about the potential of PNS could be applicable to other cases of international organizations or multilateral environmental agreements

**Answer:** The case of ISA’s Secretariat is not unique, the section that describes the role of the Secretariat is provided to show the context in which we position our main argument. Otherwise, one could also argue that the policy advice is in the hands of experts and not Secretariats. Therefore, we felt it was important to show why we focus our attention on the Secretariat. Also, choosing a specific level of analysis allows one to tackle a specific context rather than making the text and the arguments too broad and too complex. We do, however, acknowledge that the reality is more complex and the ISA Secretariat is only one component in the policy advice space. Yet, we would like to emphasize the value of the works cited to show the complexity of the Secretariat’s role. The works of Bosco and colleagues (2023) and Billet and colleagues (2023) should not be understated.  

 Just as ecosystem-based management is a helpful framework, a knowledge-ecosystem management framework is needed to manage science and expert advice in the otherwise messy context of intergovernmental organizations. One good example of a “messy” context comes from the comparative work between IPCC and IPBES:  

 Borie, M., Mahony, M., Obermeister, N., & Hulme, M. (2021). Knowing like a global expert organization: Comparative insights from the IPCC and IPBES. Global Environmental Change, 68, 102261.  

 Our main thesis is therefore that PNS is a useful framework, and could be seen as a knowledge-ecosystem management. PNS is useful for other intergovernmental bodies and contexts. It is widely discussed in the literature since the 90s. The cited Kovacic et al. (2019) work is only one recent example (with a focus on Europe and the EU Commission). The most recent work by Min Hyung Kim and Dorothy Jane Dankel (2025) presents PNS in the context of the International Council for the Exploration of the Sea (ICES):  

 Kim, M. H., & Dankel, D. J. (2025). Are We Ready To Be Wrong? Extended Peer Community for Quality Science-Advice in Uncertainty. Futures, 103520.   

 Yet, just like the reviewer suggested, ISA does not get as much scholarly attention as other better-known intergovernmental organizations such as the above-mentioned IPCC or IPBES. Moreover, IPCC and IPBES reports explicitly acknowledge the value of knowledge diversity. For example, the newly released IPBES report (Dec 2024) mentions “knowledge” 84 times, among the examples are “traditional knowledge”, “local knowledge” and “knowledge co-creation.” By contrast, ISA’s Secretariat does not yet have a comparable rhetoric in its documents. The analysis of this, however, is beyond the scope of our manuscript.   

 We will integrate these context notes into the updated manuscript.

**Question:**

 3. Several notions and concepts are mentioned in the manuscript. However, in some cases, no or limited explanation is provided. It would be important to briefly elucidate the meaning of these concepts for readers who are not familiar with the context (e.g. ‘blue growth wave’). In addition to the key concepts that will be discussed in the next points, I believe that especially two notions deserve clarification: a) ‘the Area’. The meaning of this notion is not straightforward and a better explanation of it should be provided at the very beginning of the paper; b) ‘transformative actions’. This expression is mentioned in central passages of the manuscript to indicate a key end goal: can the authors explain what they mean by it?

**Answer:** We will clarify the “blue growth” concept in an updated version of the manuscript.   

 As it appears in the first paragraph, the Area is mentioned as follows: “the international seabed – the Area.” The Area is “the international seabed.” This is the legal meaning of the term and this is the meaning as used in the ISA rhetoric and the academic literature. We will clarify the legal meaning of “The Area” in the new draft.  

 We will clarify “transformative actions” in the manuscript. In short, we echo the global academic and non-academic use of it to mean “just” transformations (this directly connects to our chosen example of justice-related literature).   

**Question:**

 4. I would invite the authors to reflect more in-depth on the nature of ‘epistemic severing’, as this concept is central to the author’s argument. Do the authors understand epistemic severing as a deliberate or inadvertent practice? Clarifying this would be particularly important also in light of the strong claims made on page 9 (“Here, we suggest that the critical negative impacts of the epistemic severing of traditional knowledge of local communities in the ISA Secretariat’s report represent a new way of making Pacific Islanders dispensable”).

**Answer:** While we do see the concept as one of the key concepts in the epistemic diversity discussions, we would like to clarify that the central argument is about PNS and the value of PNS. As mentioned earlier, applying the PNS framework does not necessarily relate to epistemic justice questions (not all exclusion of knowledge arises from epistemic severing). It is the use of PNS that invites acknowledgement of a broader range of expertise as we show with the example of the ecosystem valuation report. In turn, “epistemic severing” becomes a central concept in the illustrative example that we explore, but not a central message in the essay.   

 We would like to argue that “epistemic severing” is not necessarily deliberate or inadvertent. We suggest that the discussions on epistemic justice are, in part, an example of privilege. Not all experts, rights-holders, and stakeholders have the privilege to acknowledge epistemic justice questions and find their own space in such a debate. It is beyond our work here to research or speculate if the particular report that we chose is an example of deliberate or inadvertent practice. The concept of severing as we see it and use it does not invite to blame, it rather invites to seek repair.   

 We also acknowledge that the discussions on epistemic justice do not mean that there is institutional capacity and political will at the ISA’s Secretariat to embrace the idea and to develop approaches to implement some associated initiatives. We also see a difference between academic and political debate and we do acknowledge that our essay is an academic essay, which limits its impact in the political space.  

 Finally, “making Pacific Islanders dispensable” does not imply that the authors of the report or the Secretariat knowingly and/or willingly create such a situation. As mentioned above, the epistemic diversity debate is, in part, a privilege and a matter of skills and expertise. Those with such skills should contribute to questioning the problematic reproduction of inequality, which is what we aim to do with this text. It is not our goal to blame someone or some governing body and we do not see any value in assigning a blame here. We will clarify these aspects in the updated manuscript.

**Question:**

 5. It is unclear what “metaphorically recalibrate” exactly means. Does it mean that the contribution of PNS would be limited to a cosmetic discursive shift? I suspect that the authors’ ambition is higher than that. If so, can the authors describe in more detail how PNS could foster a knowledge ecosystem in practice? In other words, what should the Secretariat do to operationalize the ideal of PNS and translate it into action? The example presented by the authors is useful in showing what the ISA Secretariat could do as far as the content of its policy documents is concerned (i.e. broadening the knowledge base of the reports and ensuring room for alternative epistemic framings therein). However, the questions remain: how could the Secretariat go about ‘epistemic repair’ in practice? Is enriching reports with inputs from various knowledges sufficient to this end? I suspect that the answers may lie in a more detailed articulation of what a ‘knowledge ecosystem-based management’ would consist of. At the moment, no such account is provided in the manuscript. Offering more clarity on this aspect may substantially increase the policy-relevance of the paper, especially if one of its aims is informing the work programme to be undertaken by the upcoming ISA Secretary-General.

**Answer:** We clarify the idea in the following part of our text:  

 “We suggest looking at the challenge as an attempt to recalibrate policy-informing instruments through PNS. How would one “recalibrate” such instruments (e.g., reports)? That is, how can policy-informing science and expertise be more attentive to, and more able to challenge hegemonic practices?”  

 In this case, by “metaphorical recalibration” we mean challenging hegemonic practices with the main focus on “policy-informing science and expertise.” This therefore is not just a cosmetic shift or a change of semantics. Since our focus is the Secretariat, we see all its policy-advice work as the focus of the PNS approach. So, reports and other outputs that relate to the policy-advice space are the aim of PNS – “knowledge ecosystem-based management.” We present it in the manuscript as follows:  

 “In the same way that ecosystem-based management is used as a framework to provide structure to deep-seabed governance and management, we suggest post-normal science as a framework for informing policy-shaping with a focus on knowledge ecosystems. Post-normal science centralizes transdisciplinarity through an extended peer community of diverse knowledge holders – a knowledge ecosystem.”   

 In the section “The post-normal science lens” we clarify: “In sum, PNS could be seen as a knowledge ecosystem-based management.”  

 In other words, we use this analogy with ecosystem-based management to help the reader see what PNS is in simple terms. Here, we assume that among our target readership the meaning of “ecosystem-based management” is relatively well-known. We also use this analogy with ecosystem-based management to show that such an approach is a norm in managing natural systems, in turn, we hint that there is nothing unusual to aim and manage knowledge systems. In sum, as we show it, ‘knowledge ecosystem-based management’ consists of PNS where the main attention to increase the quality of policy advice is focused on the peer community.   

 However, as in the previous response, so too here, we are aware that this is an academic essay and while we do hope that it can help improve policy advice quality at ISA, it does not mean we can realistically expect that there is a will and capacity to change the internal work culture just because we wrote this essay. We see PNS as something that requires political will and voluntary engagement besides any structured list of steps that intergovernmental bodies could implement (which are always context-specific).  

**Question: ** 

 6. Besides the purported benefits of PNS (“improve the public perception of the Secretariat’s work, nurture trust between interested parties, and allow public discourse on the deep seabed to become less contentious”), have the authors considered the possible challenges that the Secretariat may encounter in implementing their proposed approach?

**Answer:** We have shortly mentioned it in the following part of the manuscript:  

 “Before we start, we anticipate one argument against routinely using the PNS lens when preparing reports is that doing so will require additional time and financing. Yet, we argue that using PNS requires only engagement with the extended peer community and its knowledge, thereby reducing the likelihood of bias.”  

 We believe that ISA has a broad range of expertise engaged in the policy context, including, but not limited to the expertise of the observers such as the Deep Ocean Stewardship Initiative (DOSI), who also write policy reports. We are not familiar with the potential challenges within the Secretariat and could only speculate what those might be. As in the previous response, we see PNS as a framework that requires voluntary engagement and a cultural shift. We hope that our article, in concert with the cited literature, could contribute to such a cultural shift. 

**Question:**

 7. Language & style. There a few grammar imperfections, including redundancies. The syntax should also be improved, as some sentences are ill-constructed or unnecessarily convoluted. Before acceptance, I recommend that the manuscript be thoroughly proofread. 

**Answer:** The authors will proofread the draft and upload a new version of the manuscript.

 Thank you again for the feedback and we hope the updated manuscript will clarify the presented argument.  

 Kind regards, 

 Aiste, Paul, Silvio, Laura, and Ola

# Reviewer report: Reviewer response for version 1

**DOI:** 10.21956/openreseurope.20429.r47321

**Published:** 2024-12-04

**Authors:** Nitin Agarwala

**Affiliations:** 1 Centre for Joint Warfare Studies, New Delhi, India

The topic of discussion is unique. However, there are some infirmities. To begin with why is the term ocean written with a capital O is not clear. Similarly, the term is deep seabed mining and NOT deep sea mining. The discussion lacks an explicit research question which is considered essential. Since this is missing, the work becomes weak. Just by saying that PNS will help decision making will not do. It needs to be justified. The authors have given an example but have not shown how PNS would / could have improved the outcome of the report / decision. The very need of PNS is not understandable for DSM and by ISA as the 4 variables for decision making are not necessarily in place for PNS to be brought to force. This thought emerges from the view that why is DSM required? For minerals!! What has happened to recycling and Circular Economy?? If one was to focus their energies towards these two, possibly the devastation of the environment could be addressed.

 Similarly, mentioning that the Oceanic states consider the ocean as their home and hence their decision and viewpoint needs to be factored in. The though is not debatable but one needs to look at the fact that Nauru which is an Oceanic state has put ISA in a bind for a forced decision (still pending) for economic gains without any consideration for the 'home'. So how can such thoughts support/ prove the argument that PNS will improve decision making. In the same breath one would say that all decisions in a human life including those made at home are politically motivated in some form or the other and not solely driven by science. Science decisions allows lesser argument as the proof of the pudding is in front. One may take Climate change as a very strong example for such decisions. The world community has accepted the impact of Climate change very late only after science gave the numbers and proof. Left to peer discussion we will still be saying 'a industry driven agenda'.

 The argument can be taken forward by saying that a paper is also a similar form of expression. The views that are being penned by this reviewer may not be in agreement with the authors or with other reviewers, so how do we then come to a decision!! It would be by merely a vote margin.

 In the present form, the article cannot be accepted as being fit for approval and needs to factor in the thoughts expressed above.

 The authors need to provide a more convincing requirement of shifting from the scientific argument to peer review argument because such a mechanism will increase subjectivity and then vote banks can be swayed either way with the right weight.

Is the argument persuasive and supported by appropriate evidence?

No

Is the work clearly and cogently presented?

No

Is the topic of the essay discussed accurately in the context of the current literature?

No

Does the essay contribute to the cultural, historical, social understanding of the field?

Partly

Reviewer Expertise:

Deep Seabed mining, Ocean Engineering, Climate Change, Marine Pollution

I confirm that I have read this submission and believe that I have an appropriate level of expertise to state that I do not consider it to be of an acceptable scientific standard, for reasons outlined above.

# Response

**Published:** 2024-12-20

**Authors:** aistė klimašauskaitė

**Affiliations:** University of Bergen, Norway

Dear Reviewer,

 We want to thank you for the speedy first review.

 As a multidisciplinary group of authors, we tried to make our work clear and accessible for the intended audience by removing subject-specific jargon and explaining specific terms where necessary. We streamlined the story to make logical connections between the argument, the literature, and the developments at ISA. We feel it is important to clarify and correct things that the readers find confusing. However, we believe that some of the misunderstandings are not possible to address in a simple rewrite and we hope that our explanations below clarify some of the issues.  

 -------------------------------------------------------

 Here is the summary of our responses to the comments (detailed comments appear below):

1. PNS is not unique as suggested and we feel it is important to stress that we build on an extensive work of the last 30+ years. The academic PNS discussions resulted in a relatively large body of works that include works in marine science.
2. The term “deep-sea mining” is used in academic literature, in the debates at the International Seabed Authority, and by the reviewer in their work as well. So, the suggestion that the term is wrong seems puzzling.
3. There is a misunderstanding regarding the form of our text. This is not an empirical research article. It is an argumentative essay. Therefore, there is no research question, there is a thesis statement instead.
4. The reviewer suggests that the recommendation for PNS “emerges from the view that why is DSM required.” Yet, at no point in the text do we question the need for DSM. Our focus and the value of PNS is to improve policy advice. It is not about answering whether DSM is needed or not.
5. The reviewer seems to suggest that the example of Nauru negates the need to improve policy-relevant advice at ISA. The reviewer also implies that since Nauru sponsors the Metals Company, the Nauruans do not see the Ocean as “home.” We suggest that these are puzzling assumptions.
6. It is unclear why the reviewer sees contributions to improve the quality of policy advice as a need to vote on which knowledge is better or more valuable. In our illustrative example, we explicitly write that assigning weight to specific values of the ecosystem services is problematic. Improving policy advice does not mean voting among transdisciplinary experts to select some superior type of value of a specific ecosystem service, it means providing adequate and quality information for policymakers about the existing values. 
7. It is confusing why the reviewer thinks PNS calls for shifting away from science. At no point in the essay do we claim so and the PNS literature does not call for it. We explicitly write in the manuscript that PNS “is not an alternative to science.” Throughout the manuscript, we show that PNS is about improving policy advice and, in particular, complementing science for policy advice with transdisciplinary expertise.

**Below are detailed responses to the reviewer’s comments.**  

**Reviewer’s comments: **"The topic of discussion is unique. However, there are some infirmities."

**Our response: **Applying post-normal science (PNS) in the context of science for policy advice is not a unique idea. We feel we demonstrated this in the manuscript under the subtitle "The post-normal science lens" where we point out the discussions on the topic since the 90s.

 If the reviewer meant that connecting PNS and marine science is unique, we suggest it is also not the case as the literature shows:

 Kim, M. H., & Dankel, D. J. (2024). Are We Ready To Be Wrong? Extended Peer Community for Quality Science-Advice in Uncertainty. *Futures*, 103520.

 Cavaleri Gerhardinger, L., Brodie Rudolph, T., Gaill, F., Mortyn, G., Littley, E., Vincent, A., ... & Colonese, A. C. (2023). Bridging Shades of Blue: Co-constructing Knowledge with the International Panel for Ocean Sustainability. *Coastal Management, 51*(4), 244-264.

 Blanchard, A., Hauge, K. H., Andersen, G., Fosså, J. H., Grøsvik, B. E., Handegard, N. O., ... & Vikebø, F. (2014). Harmful routines? Uncertainty in science and conflicting views on routine petroleum operations in Norway. *Marine Policy*, *43*, 313-320.

 Polejack, A. (2021). The importance of ocean science diplomacy for ocean affairs, global sustainability, and the UN decade of ocean science. *Frontiers in Marine Science, 8*, 664066.  

**Reviewer’s comments: “**To begin with why is the term ocean written with a capital O is not clear.”

**Our response: **We did not explain our use of an uppercase "O" at the beginning of "Ocean." We will add a clarification in a new version of the article. Overall, we wanted to emphasize the marine environment as one global and interconnected body of water, which was first inspired by the famous "Spilhaus projection":

 Spilhaus, A. F. (1942). Maps of the whole world ocean. *Geographical Review, 32*(3), 431-435.

 When one refers to a specific region, it is appropriate to use an uppercase letter. See for example "the South", "the North", or in the present case "the Ocean". Some marine scientists choose to use capital “O” to normalize the idea that planet Earth is actually planet Ocean (due to the relative size and coverage of the Ocean). Others use capital O to normalize the appreciation and the value of the Ocean for all humanity. Capitalization of “O” is becoming more popular in the academic literature as well:

 Dankel, D. J., Aasly, K., Carić, H., Ellefmo, S. L., Gaspers, A., Hixson, R., ... & Cowan, E. (2023). Ocean sectors: Case studies of human activity in the Ocean-based economy. *In Oceans and Human Health* (pp. 531-546). Academic Press.  

**Reviewer’s comments:** “Similarly, the term is deep seabed mining and NOT deep sea mining.”

**Our response: **The criticized use of "deep-sea mining" in place of "deep-seabed mining" is puzzling. We would like to draw attention to the reviewer's own body of work where they:

 1) Use the term “deep-sea mining” in the body of the text: Agarwala, C. D. N. (2019). *Deep seabed mining in the Indian Ocean: economic and strategic dimensions*. National Maritime Foundation.  

 2) Frequently cite other papers which use the term "deep-sea mining" rather than "deep-seabed mining" and quote the term in their text, for example:

 Agarwala, N. (2023). Using robotics to achieve ocean sustainability during the exploration phase of deep seabed mining. *Marine Technology Society Journal, 57*(1), 130-150.

 We also want to point out that the term “deep-sea mining” is used more frequently in the literature than “deep-seabed mining”. A search on Google Scholar found approximately 16,800 entries when the search term was “deep-sea mining” compared to approximately 8,880 entries when “deep-seabed mining” was used as a search term.

 Finally, we would like to stress that both of the terms are used by the International Seabed Authority. A brief read of the International Seabed Authority’s website ( https://www.isa.org.jm/), including one of their recent publications ( https://www.isa.org.jm/wp-content/uploads/2024/12/DEEP-DIVE-One-year-of-Deep-Dive-%E2%80%93-an-immersive-journey-into-the-multidisciplinary-knowledge-of-the-deep-sea.pdf) shows both terms are used, seemingly interchangeably.

 The term “deep-sea mining” could be seen as more specific because deep-sea mining operations would presumably happen both on the seabed and in the water column. This would include the generation of mining plumes and midwater plumes as part of the operations:

 Muñoz-Royo, C., Peacock, T., Alford, M. H., Smith, J. A., Le Boyer, A., Kulkarni, C. S., ... & Ju, S. J. (2021). Extent of impact of deep-sea nodule mining midwater plumes is influenced by sediment loading, turbulence and thresholds. *Communications Earth & Environment, 2*(1), 148.

 In the updated manuscript, we will add a footnote clarifying the wording in the academic literature and policy jargon that exists in the context of the topic.  

**Reviewer’s comments:** “The discussion lacks an explicit research question which is considered essential. Since this is missing, the work becomes weak. Just by saying that PNS will help decision making will not do. It needs to be justified.”

**Our response: **We would like to emphasize that this is not an empirical paper. This text was submitted as an essay and is marked as an essay on the ORE platform. As an argumentative essay, it has a thesis statement and not a research question. Also, commentary writing, which our essay falls under, frequently does not have a research question. Rather, it is a discussion of what applying one tool to a current debate can accomplish.

 We provide the thesis statement immediately in the abstract:

 “In short, we argue that post-normal science can help metaphorically recalibrate policy-shaping instruments (the practice) so that decision-making processes and policy agendas are implemented for transformative actions.”

 This thesis is echoed in the very first paragraph as well: “Therefore, we propose a policy-relevant framework to be used when stakes are high and values contested.”

 In the updated manuscript, we will clarify this by adding an explicit phrase: “Here is our thesis:”

 Finally, the literature on PNS and science for policy advice is very broad and falls outside of the scope of our essay, which focuses on the use of PNS. As a concrete example of using PNS, we choose epistemic justice-related literature to show how it contributes to the quality of valuation studies. For example, we cite theoretical literature (e.g., Alcoff, Hau’ofa, Massimi) and empirical literature (e.g., Mulalap, Villagomez and Johnson) to show how epistemic injustices operate and how they can be disrupted. It is unclear why the reviewer thinks such works add no value to policy discussions. In other words, the value of PNS lies not only in its theoretical claims but also in what practically such a framework can add to a given policy context – this is the part that we chose to highlight with our example of the ecosystem valuation report. So, we do not merely say that PNS would better inform decisions, we give an illustrative example of how it could work.  

**Reviewer’s comments:** “The authors have given an example but have not shown how PNS would / could have improved the outcome of the report / decision. The very need of PNS is not understandable for DSM and by ISA as the 4 variables for decision making are not necessarily in place for PNS to be brought to force. This thought emerges from the view that why is DSM required? For minerals!! What has happened to recycling and Circular Economy?? If one was to focus their energies towards these two, possibly the devastation of the environment could be addressed.”

**Our response: **We would like to stress that we do not recommend the need for PNS for DSM. We recommend PNS for policy advice. ISA uses various reports and expert insights for policy advice purposes. A suggestion that such advice could be improved is not controversial in itself and it is unclear why the reviewer does not find it understandable.

 It is puzzling to read that a text clearly focused on improving the quality of policy advice outputs is perceived as questioning “why is DSM required”. At no point in the text do we comment on whether DSM is required or not required. Similarly, we do not explore recycling or circular economy. The questions lie outside the scope of our original discussion. We agree that circular economy and recycling should be utilized, but this is not the subject of our article.  

**Reviewer’s comments:** “Similarly, mentioning that the Oceanic states consider the ocean as their home and hence their decision and viewpoint needs to be factored in. The though is not debatable but one needs to look at the fact that Nauru which is an Oceanic state has put ISA in a bind for a forced decision (still pending) for economic gains without any consideration for the 'home'. So how can such thoughts support/ prove the argument that PNS will improve decision making.”

**Our response: **We believe the reviewer implies the two-year rule invoked by Nauru at ISA. This relates to the trigger to finalize the Mining Code within two years of the invocation (the two years expired on 9 July 2023) or the need to consider applications for extraction before the Mining Code is finalized. While the reviewer suggests the decision is still “pending”, ISA made a decision (source: https://www.isa.org.jm/news/isa-council-closes-part-ii-of-its-28th-session/):

 “The so-called “two-year rule” expired on 9 July 2023. However, no application for a plan of work has been received to date.

 The Council also adopted a decision relating to the understanding and application of section 1, paragraph 15, of the Annex to the Agreement relating to the Implementation of Part XI of UNCLOS including steps to be taken if an application for a plan of work for exploitation is submitted before the Council has completed the rules, regulations and procedures (ISBA/28/C/25).”

 Also, we would like to stress that Nauru’s position does not negate the meaning of the Ocean as the home for the Pacific Islanders and for the Nauruans themselves. It is unclear why the reviewer implies that it does.

 Nauru, the sponsoring state of the Metals Company (TMC), is an example of “sponsorship of convenience.” This has been discussed in the literature and it is about the problematic behavior of private actors (in this case TMC) who engage with some of the island states with questionable intentions, including hoping for more lax accountability and encroaching on the areas for exploration in the international seabed – the Clarion Clipperton Zone – that are reserved for such island states and not for private actors. This has been discussed in academic as well as non-academic literature. Singh (2021) has a clear description of these issues:

 Singh, P. A. (2021). The two-year deadline to complete the International Seabed Authority’s Mining Code: Key outstanding matters that still need to be resolved. *Marine Policy, 134,* 104804.

 We cite Holst (2023) in our manuscript, and we would like to quote her here to show how problematic is the relationship between Nauru and the Metals Company:

 “Apart from the fact that it is questionable to what extent this role ultimately benefits the sponsoring state and its population as considered above, the enhanced capacity is to a not insignificant extent directly beneficial to the contractor. Shortly after the small island sponsoring states mentioned above had applied for exploration contracts, the European Union (EU) offered development assistance to Pacific Island states, including Nauru, to help them improve the governance and management of deep sea minerals and draft national legislation. As a result, NORI ‘not only [gained] access to areas reserved for exploration and exploitation by developing states; it also is the indirect beneficiary of development assistance by the EU aimed at enabling Nauru to meet its obligations as a sponsoring state’. In deep sea exploration, that is one small step for Nauru, one giant leap for NORI – rather than humankind.”  

**Reviewer’s comments: “**In the same breath one would say that all decisions in a human life including those made at home are politically motivated in some form or the other and not solely driven by science.”

**Our response: **Political decisions at ISA are surely “politically motivated in some form or the other and not solely driven by science”. We do not claim otherwise and we show how the Secretariat shapes policy-making through various outputs, (including various reports like the one we cite) and how those outputs are used within ISA. We have cited multiple relevant papers to illustrate it. Does the reviewer suggest that such outputs are not used or useful for policymakers at ISA?  

**Reviewer’s comments:** “Science decisions allows lesser argument as the proof of the pudding is in front. One may take Climate change as a very strong example for such decisions. The world community has accepted the impact of Climate change very late only after science gave the numbers and proof. Left to peer discussion we will still be saying 'a industry driven agenda'.”

**Our response: **After an extensive discussion between authors, we do not know what the reviewer means when writing about the proof of the pudding being at the front. Our text is about improving policy advice at ISA. We do not touch on the question of whether or not the global community accepts science or science advice.

 We would like to address the following quote: “Left to peer discussion we will still be saying 'a industry driven agenda'.” We would like to explicitly state that at no point did we suggest that the Secretariat’s report we took as an example indicates an “industry driven agenda.” In the illustrative example (the valuation report (Brander & Guisado Goñi, 2023)), we discuss knowledge and detail the problem of epistemic hegemony and severing. The example has nothing to do with industry or industry agenda and we do not imply so. The report we chose to explore as an example (Brander & Guisado Goñi, 2023) shows a problem of epistemic hegemony, not an industry agenda.  

**Reviewer’s comments:** “The argument can be taken forward by saying that a paper is also a similar form of expression. The views that are being penned by this reviewer may not be in agreement with the authors or with other reviewers, so how do we then come to a decision!! It would be by merely a vote margin.”

**Our response: **There seems to be a misunderstanding of what is a view and what is knowledge. We do not discuss people’s views, we discuss transdisciplinary knowledge for the purpose of policy advice.

 There is a fundamental misunderstanding here about the value of acknowledging important knowledge holders and their knowledge. It is unclear why the reviewer thinks that all combined knowledge needs to be voted on to select the winning knowledge or data source. Our argument about the problem of weighing knowledges and their value warns exactly about such approaches:

 “Next, we would like to go back to the question: What does it mean to find ecosystem services that are “key” and of “high economic value”? The above discussion was focused on finding. Here, we would like the reader to turn attention to the word “key.” What does it mean to present values as competing and to position some as key while leaving others as potentially low-key or missing, as we showed above? Does it mean we can make lists of values with competing weights? Assigning a monetary value is heavily criticized in the literature, and the report authors acknowledge such criticism (Brander & Guisado Goñi, 2023). It is interesting to note that while the authors do not assign a monetary value as such (due to study limitations), they do seem to follow the monetary logic that some ecosystem services have more value than others. Who chooses to assign weight? Can a report improve its quality by acknowledging the alternative choices and refusing to weigh the values? Would policymakers be better informed by acknowledging alternative choices or the biases of experts chosen to assign the weight of values? We leave these questions open for future discussions.”

 Finally, in the context of the chosen example (the report by Brander & Guisado Goñi (2023)), before any assessment of what is the value of ecosystem services, one needs to know what those services are – all the different values. The reviewer, however, seems to suggest there is no need to know what those diverse ecosystem services and their values are. They also seem to suggest that improving the quality of information in policy advice is not needed. To us, these claims are puzzling.   

**Reviewer’s comments: **“The authors need to provide a more convincing requirement of shifting from the scientific argument to peer review argument because such a mechanism will increase subjectivity and then vote banks can be swayed either way with the right weight.”

**Our response:** This is a fundamental misunderstanding of PNS and the manuscript. We specifically state that PNS is not about shifting from science to PNS. It is about complementing science with PNS approaches as we write in the manuscript:

 “It is important to note that PNS is not an alternative to science or a different kind of science, but rather different conditions of the science for policy context (Saltelli et al., 2020). It is a framework by which science informs policy under complex conditions. Reaching quality outcomes in such situations requires rich knowledge from diverse sources.”

 Also, we do not suggest “peer review”, we talk about a well-known PNS component – a peer community or a community of transdisciplinary peers in providing policy-relevant advice.

 All these comments from the reviewer show that there is a fundamental misunderstanding of what a peer community is. It is not about agreement between peers or a vote between peers. It is about acknowledgement and inclusion of diverse knowledges in policy advice where stakes are high and values in dispute. Similarly, the decisions at ISA are not made by scientists or civil society – they do not have a right to vote. The decisions are taken by states. PNS is a framework for quality policy advice, PNS is not about agreements or disagreements among experts or politicians.  

**The following are ORE questions for reviewers to which the reviewer gave responses:**  

**ORE:** “Is the topic of the essay discussed accurately in the context of the current literature?”

 The reviewer suggests “No.”

**Our comment:** It is entirely unclear why the answer is "no" as the reviewer neither criticized any of the literature used by us nor recommended any literature which should be added.  

**ORE:** “Is the work clearly and cogently presented?”

 The reviewer suggests “No.”

**Our comment:** We would like to argue that the work is clear. We start by introducing the broader context at ISA. We then move to detail the role of the Secretariat in shaping policy decisions through various science and expert reports and outputs. We continue by presenting the PNS lens. Finally, we apply the PNS lens to a specific example. It is unclear to us, how after reading the text, the reviewer decided that, for example, PNS is a call to move away from science.  

**ORE:** Is the argument persuasive and supported by appropriate evidence?

 The reviewer suggests “No.”

**Our comment:** It is unclear why the reviewer finds the argument not persuasive. As explained above, there seems to be a fundamental misunderstanding regarding the science and expert advice at ISA, which we describe in the section “The Secretariat as a key body in policy-shaping at ISA.” It seems that the reviewer is not convinced that improving policy advice is something of value. The appropriate evidence, we argue, is all the literature presented to show how ISA’s Secretariat uses science and expert inputs. Following from that, we chose one specific report as an example, where we used diverse literature to support our argument. The reviewer has not pointed out any flaws in the evidence cited, nor have they cited any evidence to dispute our arguments.    

 We wish to thank the reviewer for their comments and hope that our replies clarify some of the misunderstandings.  

 Kind regards, Aiste, Paul, Silvio, Laura, and Ola

 ------------------------------------------------------------------
